# Interlayer Spacing Control of MoS_2_ with Covalent Thiol Functionalization: Understanding Structure and Electrochemistry from Experiments and Simulation

**DOI:** 10.1021/acsnano.5c07717

**Published:** 2025-10-02

**Authors:** Jaehoon Choi, Kyeonghyeon Nam, Yoga T. Malik, Robert Leiter, Maider Zarrabeitia, Christoph Scheurer, Simon Fleischmann

**Affiliations:** 1 557388 Helmholtz Institute Ulm (HIU), Helmholtzstraße 11, 89081 Ulm, Germany; 2 150232 Karlsruhe Institute of Technology (KIT), 76021 Karlsruhe, Germany; 3 28259 Fritz Haber Institute of the Max Plank Society, 14195 Berlin, Germany; 4 IET-1, Forschungszentrum Jülich, 52425 Jülich, Germany

**Keywords:** Molybdenum disulfide, Transition metal dichalcogenides, Covalent functionalization, Interlayer spacing engineering, Electrochemical energy storage, Lithium-ion intercalation

## Abstract

Molybdenum disulfide (MoS_2_) is an increasingly investigated two-dimensional electrode material for electrochemical energy storage and conversion. Strategies to increase its interlayer spacing are emerging and have been shown to improve ion intercalation capacity and kinetics. This work explores covalent thiol functionalization for controlling MoS_2_ interlayer spacing. Using a hydrothermal bottom-up synthesis, dithiolated molecules can be directly incorporated into the MoS_2_ lattice to act as pillars. Using a comprehensive combination of experiments and simulation, we investigate the influence of dithiol pillar loading on the emerging structure, pillar–host interactions, and electrochemistry. Our results reveal clustering of pillars at low loading, leading to an inhomogeneous interlayer expansion. At high pillar loading, the formation of defective bonding configurations with excess sulfur is observed. Interlayer expansion leads to an increased electrochemical Li^+^ storage capacity with a maximum of 1.43 Li^+^ per MoS_2_. However, dithiols occupy storage sites and impede Li^+^ transport within the interlayer space, leading to unfavorable performance at high pillar loading. This underlines the importance of carefully adjusting the density of nanoconfined pillar molecules within the interlayer space. Overall, the work comprehensively analyzes covalent dithiol functionalization of transition metal dichalcogenide-based electrode materials, offering valuable insights for the design of advanced energy materials.

## Graphical abstract

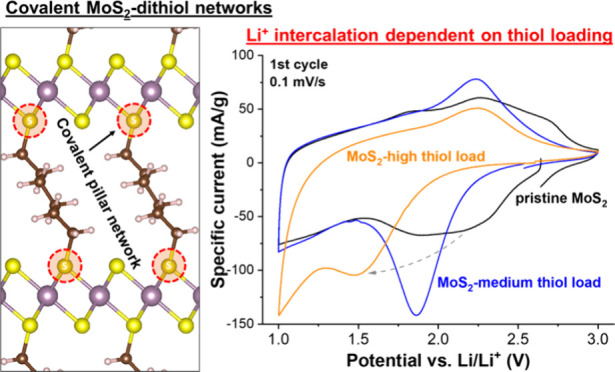

Two-dimensional (2D) materials have attracted significant attention due to their unique structural and functional properties. Among these, transition metal dichalcogenides (TMDs) such as molybdenum disulfide (MoS_2_) have emerged as promising candidates for next-generation energy storage systems. In its nanostructured form, MoS_2_ has been shown to exhibit favorable pseudocapacitive ion intercalation properties, that is, exhibiting a capacitor-like voltage profile with fast and electrochemically reversible kinetics based on Faradaic reactions.^1,2^ Since layered materials serve as host structures for electrochemical ion intercalation, where ions are stored between the layers, the nanoconfinement properties of the interlayer space play a pivotal role in determining the overall ion intercalation performance.^3^ Importantly, increasing the interlayer spacing in transition metal oxides has been shown to simultaneously influence ion intercalation kinetics, the maximum storage capacity, as well as the charge storage mechanism itself.^4^ There is a need for a detailed understanding of how nanoconfinement properties impact on ion diffusion paths and storage sites.

MXenes represent another prominent class of 2D materials whose remarkable success in electrochemical applications can be largely attributed to their highly tunable nanoconfinement chemistry and geometry.^5,6^ This tunability allows for the precise engineering of ion transport pathways and charge storage mechanisms.^7^ To translate such versatility to other 2D systems like MoS_2_, there is an urgent need to investigate methods that can tailor its nanoconfining interlayer environment and broaden possible interlayer chemical compositions.

In our previous work, we demonstrated that simultaneously manipulating the crystallite size and the interlayer distance of MoS_2_ via the incorporation of hexanediamine (HDA) pillars led to improved Li^+^ intercalation kinetics.^8^ However, a significant limitation was identified: HDA does not form strong bonds with the MoS_2_ lattice. This weak interaction may compromise the long-term stability of the pillared architecture and lead to pillar dissolution upon long-term cycling, as the pillars are not sufficiently anchored to the host lattice.

To overcome this crucial bottleneck, the present study introduces hexanedithiol (HDT) as an organic pillar capable of stronger interaction with the MoS_2_ host. HDT is characterized by thiol (−SH) groups that, based on prior investigations primarily on monolayers, can form robust covalent bonds with MoS_2_.^9^ For example, such studies have shown that thiol molecules react with defect sites and sulfur vacancies in MoS_2_ to establish stable covalent linkages.^10^ Similarly, ligand conjugation approaches have successfully anchored thiol-functionalized molecules to chemically exfoliated MoS_2_, reinforcing its structural integrity.^11^ Moreover, covalent functionalization via thiol chemistry has been demonstrated to modulate the electronic properties of MoS_2_ effectively and tailor the performance of MoS_2_-based devices.^12–15^

By integrating HDT during hydrothermal synthesis, we establish a covalent network between the organic pillars and the MoS_2_ layers. We hypothesize that HDT-pillared MoS_2_ based on covalent networks will exhibit modified electrochemical properties compared to, for example, previously established amine-based pillaring architectures. Hence there is an urgent need for understanding the structure and electrochemistry of such covalently pillared host materials.

A further key parameter governing the performance of pillared materials is the density of the pillars. Previous studies on pillared systems have underscored that a lower pillar density can create more accessible diffusion paths, thereby enhancing ion mobility and storage capacity.^16^ In MoS_2_, we expect that controlling the density of HDT pillars is crucial to fine-tune the diffusion pathways and storage sites available for electrochemically intercalating ions.

In the present study, we systematically investigate the properties of covalently pillared MoS_2_ host materials, including the influence of HDT pillar density on the nanoconfinement properties and the resulting Li^+^ intercalation behavior. Detailed structural characterization reveals the evolution of the nanoconfinement properties of MoS_2_ as a function of pillar density, allowing to formulate optimized structural models. By correlating the pillar loading with changes in ionic mobility and storage capacity through a combination of experiments and simulation, we deepen the understanding of the design principles for pillared electrode materials. The strategy of covalent pillaring not only advances the fundamental knowledge of interlayer engineering but also opens new avenues for the development of stable and efficient energy storage materials.

## Results and Discussion

### Structural Characterization of MoS_2_-Based Materials

Figure 1A schematically outlines our proposed materials design strategy, in which 1,6-hexanedithiols (HDT) are directly incorporated into the MoS_2_ lattice where they act as organic pillar molecules that modify the nanoconfinement environment. The density of pillars is controlled by varying the molar ratio of pillar to Mo during hydrothermal synthesis between 0.2 and 5. The motivation behind this approach is to obtain a pillared MoS_2_ structure with (1) covalent networks between host and pillar molecules and (2) a highly tunable pillar loading. This is a significant evolution from previous approaches that employed ionic pillars that weakly interact with the host lattice and exhibited limited variability in pillar loading.^4,8,17^ The structural characterization is focused on determining how varying the HDT loading affects the resulting pillared architecture and analyzing the bonding character between HDT pillars and the MoS_2_ lattice.

**Figure 1:**
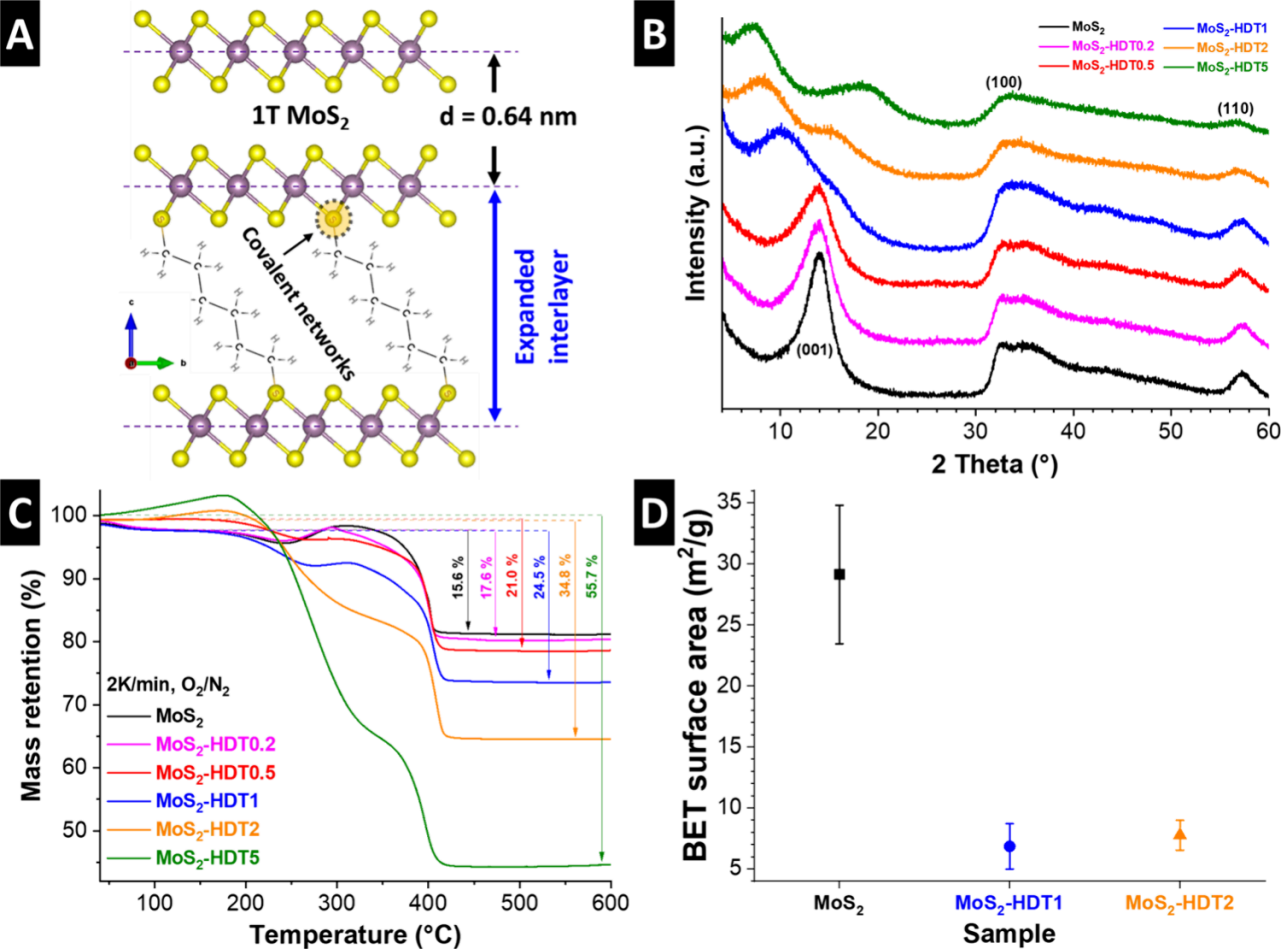
(A) Schematic illustration of the proposed formation of covalent networks between HDT pillars and MoS_2_. (B) XRD patterns of the pristine and HDT-pillared MoS_2_. (C) TGA curves and (D) BET specific surface area of MoS_2_-based samples.

The structure of hydrothermally synthesized, MoS_2_-based materials is initially investigated using X-ray diffraction (XRD). The diffractograms of pristine and HDT-pillared MoS_2_ materials with various HDT loadings are shown in Figure 1B. The formation of pristine MoS_2_ is verified by the broad reflexes centered at ca. 13.8° and 32.5° 2 Theta corresponding to the (001) and (100) planes of 1T-MoS_2_, respectively, in line with previous work.^8^ The addition of HDT in increasing quantities from 0.2 to 5 (molar ratio between pillar molecules to Mo during synthesis) causes changes in the diffractograms, predominantly related to the position and shape of the (001) signal representative of the interlayer spacing. With increasing HDT pillar loading, there is a progressive change of the (001) signal. Starting with the formation of a shoulder toward lower diffraction angles for samples with low HDT loading (MoS_2_-HDT0.2 and MoS_2_-HDT0.5), there is a significant shift toward lower diffraction angles for all samples with higher HDT loading. The shift and very broad shape of the (001) reflex is indicative of an uneven increase of the MoS_2_ interlayer spacing in pillared materials with medium HDT loading. For high pillar loadings in MoS_2_-HDT2 and MoS_2_-HDT5, the (001) signal shifts further and sharpens again, indicative of higher structural order and an evenly increased interlayer spacing of 1.1 and 1.3 nm, respectively. Moreover, we observe the formation of an additional broad diffraction signal at around 15° for MoS_2_-HDT2 and 18° 2 Theta for MoS_2_-HDT5 that can neither be related to the MoS_2_ intralayer structure, nor to higher order interlayer spacing. Therefore, we hypothesize that the origin of the signals is related to the ordering of the organic chains of HDT pillars within the interlayer galleries, which decrease in characteristic distance at the highest HDT loading in MoS_2_-HDT5.

The findings indicate that HDT-pillaring of MoS_2_ is increasingly expanding the interlayer spacing with higher loading. The uneven expansion for low and medium pillar loadings indicates that pillars preferentially form domains, while some MoS_2_ regions remain unpillared (the thermodynamic reasons are further elucidated in simulation section). Only for high loadings, the entire MoS_2_ structure becomes pillared by HDT.

The density of HDT in pillared MoS_2_ samples is quantified using thermogravimetric analysis (TGA). The mass loss under an oxidative atmosphere (O_2_/N_2_) is recorded up to 600 °C (Figure 1C). At elevated temperatures, pristine MoS_2_ converts to MoO_3_ under the release of SO_2(*g*)_, leading to a theoretical mass loss of 10%. Note that small mass gains at intermediate temperatures have been observed before and attributed to the formation of intermediates like MoO_2_, which then further oxidize to MoO_3_.^18^ The presence of significant sulfur vacancies is unlikely though, given the elemental composition results, vide infra. The excess mass loss is therefore assigned to burned-off HDT pillar molecules, allowing to quantify the content in the materials as MoS_2_-(HDT)_0.10_, MoS_2_-(HDT)_0.15_, MoS_2_-(HDT)_0.20_, MoS_2_-(HDT)_0.40_, and MoS_2_-(HDT)_1.10_ for the samples MoS_2_-HDT*x* with *x* = 0.2, 0.5, 1, 2, and 5, respectively. The results indicate that the pillar density is successfully varied from a sparsely pillared to a fully crowded interlayer space.

After covalent pillaring with HDT, the Brunauer–Emmett–Teller (BET)^19^ specific surface area decreases compared to the pristine MoS_2_ sample (Figure 1D). The N_2_ sorption isotherms of the MoS_2_-based samples resemble Type IV (Figure S1), indicative of mesoporous materials, with H3/H4-type hysteresis loops that have been described for aggregates of plate-like particles.^20^ The findings indicate that covalent pillaring increases the interconnectivity of the MoS_2_ sheets, aligning with microscopy observations, vide infra.

The structural properties of the materials are further elucidated using electron microscopy. Scanning electron micrographs of all samples show similar morphology across all samples, consisting of agglomerates of nanosized flakes (Figure S2). This demonstrates that the hydrothermal process followed by freeze-drying ensures consistent particle morphology across samples, allowing for direct comparison of their electrochemical properties later. However, at the nanoscale, differences in the structure of the primary flakes are observed in each sample through transmission electron microscopy (TEM). In Figure 2A, pristine MoS_2_ exhibits a flake-like morphology with a lateral size of approximately 50 nm. Lattice fringes show an interlayer spacing of about 0.67 nm. Three rings are observed in the selected area electron diffraction (SAED), corresponding to the (001), (100), and (110) planes. The *d*-spacing calculated from the first SAED ring is 0.64 nm, while the in-plane lattice spacings for the (100) and (110) planes are 0.25 and 0.15 nm, respectively, which is in agreement with XRD results. Pillared MoS_2_ samples with low to medium HDT-loading (MoS_2_-HDT0.2, MoS_2_-HDT0.5, MoS_2_-HDT1; Figure 2B–D) show similar flake morphologies, but regions with expanded interlayer spacing observed in high-resolution images. It can be seen that the expansion is highly irregular. The (001) ring in the corresponding SAED patterns remains most prominent at the same position as in the pristine MoS_2_, but SAED features with smaller radii can be seen particularly for MoS_2_-HDT1, indicating regions of further interlayer expansion. The results confirm XRD results also on a nanoscale, demonstrating that the HDT pillars form domains and partially expand the MoS_2_ layers, while nonexpanded MoS_2_ is retained in some regions. In contrast, HRTEM of densely pillared MoS_2_-HDT2 and MoS_2_-HDT5 (Figure 2E,F) shows larger interlayer spacings also in agreement with XRD. The related (001) rings are no longer visible in the SAED patterns due to the beam stopper. Meanwhile, the reflections corresponding to the in-plane structure remain largely unaffected by the insertion of pillars, as no significant changes are observed in (100) and (110) planes for all pillared samples. An elemental mapping using energy-dispersive X-ray spectroscopy (EDX) in STEM mode (Figure S3) depicts the homogeneous distribution of molybdenum, sulfur, and carbon in the as-synthesized samples.

**Figure 2:**
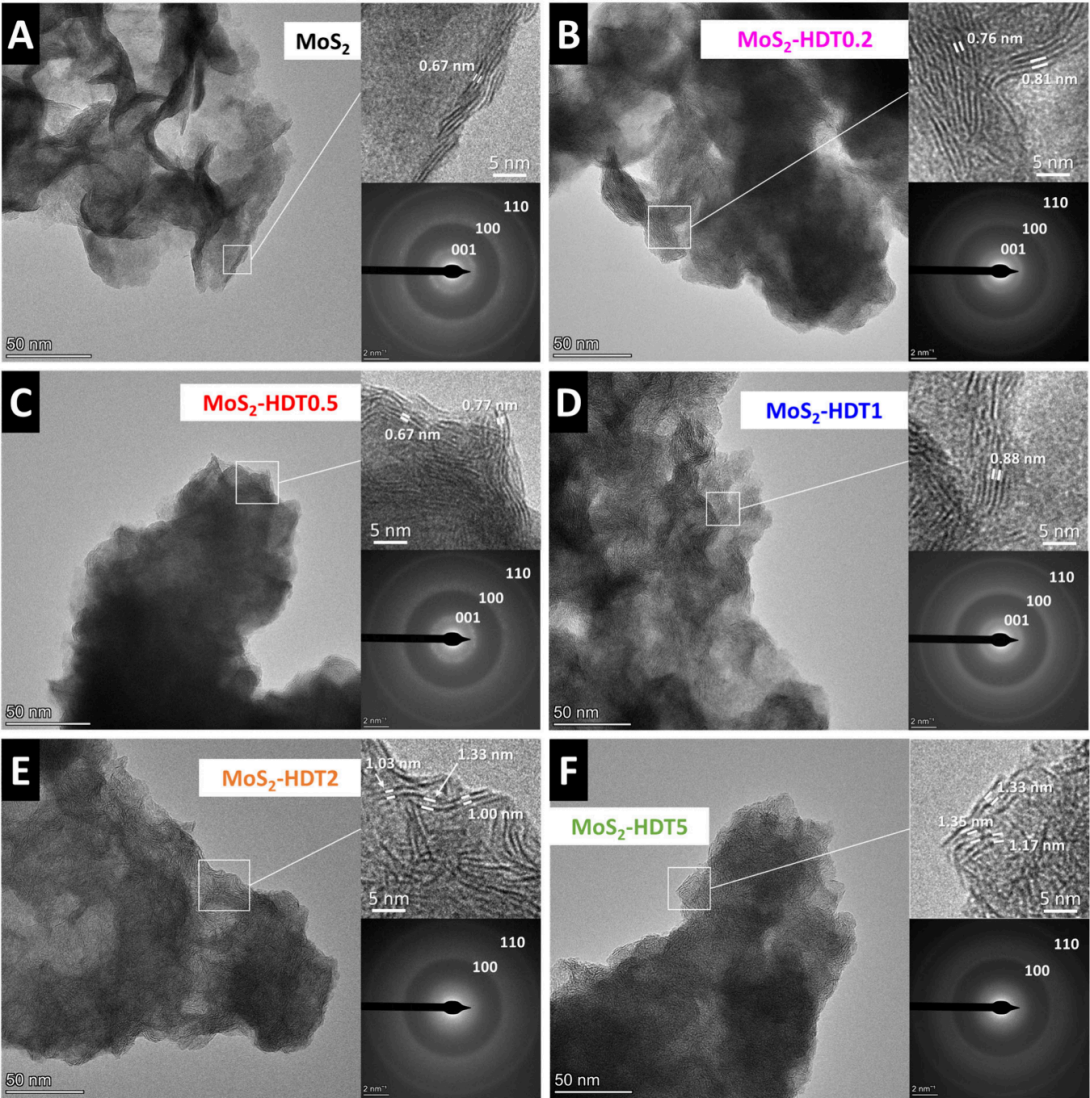
(HR)­TEM images and SAED patterns of (A) the pristine MoS_2_ and (B–F) HDT-pillared MoS_2_ samples.

In the following, the bonding character between the MoS_2_ host lattice and HDT pillars is examined with spectroscopic methods. Based on previous work on thiol-functionalized MoS_2_ monolayers,^10,11^ we hypothesize that HDT can form chemical bonds with the MoS_2_ lattice yielding a covalent network. First, Fourier-transform infrared (FTIR) spectroscopy is employed to establish the spectroscopic signature of the HDT molecule and analyze how it changes upon functionalization in the MoS_2_ lattice. Pure HDT molecules exhibit typical signatures of C–H stretching vibrations at around 2850–2960 cm^–1^ and a distinct peak corresponding to the S–H stretching vibration at 2554 cm^–1^ (Figure 3A).^21^ In HDT-pillared MoS_2_ samples, the C–H stretching vibrations are still visible and increase in intensity for increased HDT loading. However, the S–H stretching vibration can no longer be detected in any of the HDT-pillared MoS_2_ samples, which indicates that the thiol functional groups undergo chemical changes, e.g., are involved in chemical bonding with the MoS_2_ lattice. Raman spectra of pristine and HDT-functionalized MoS_2_ samples show characteristic signatures at around 150 and 330 cm^–1^ (Figure 3B), corresponding to the J_1_ and J_3_ modes of the metallic phase MoS_2_. The absence of *E*_2_^1^ *g* (usually around 379 cm^–1^) and A_1g_ (405 cm^–1^) signals characteristic for the 2H-phase^22^ is supportive of the formation of 1T-MoS_2_ in all our samples. It should be noted that the Raman measurements are conducted with a low laser power to avoid the decomposition/carbonization of HDT molecules during the measurement.

**Figure 3:**
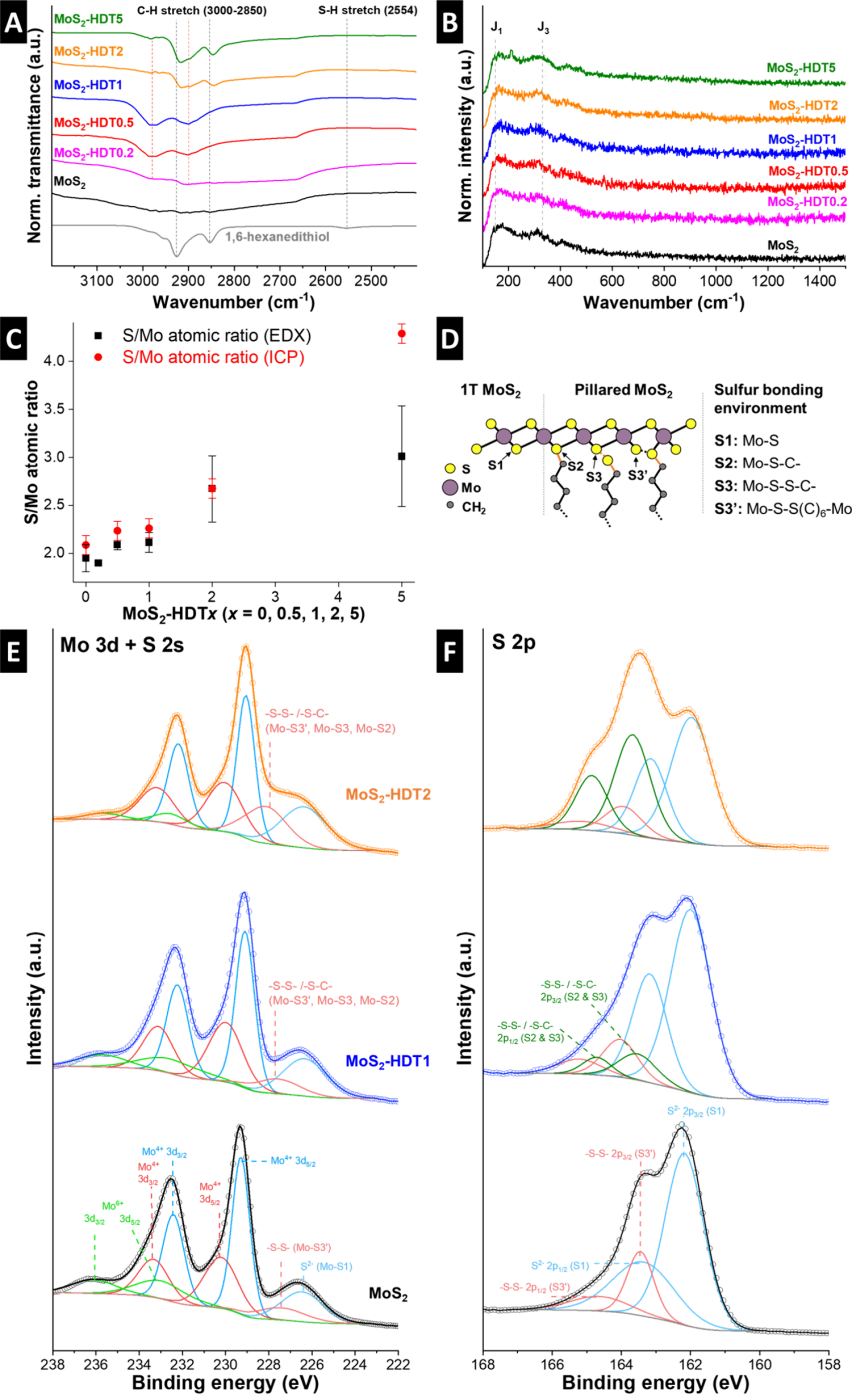
(A) FTIR and (B) Raman spectra of all samples. (C) Elemental composition of all MoS_2_-based samples from ICP-OES and STEM-EDX. (D) Proposed structural configurations illustrating sulfur atoms from pristine MoS_2_ (S1) and those corresponding to nonideal MoS_2_, such as Mo-S-C- (S2), Mo-S-S-C- (S3), and Mo-S-S-Mo (S3′). XPS spectra of (E) Mo 3d and S 2s and (F) S 2p photoelectron regions of pristine (black), MoS_2_-HDT1 (blue), and MoS_2_-HDT2 (orange) samples.

The results of Raman and FTIR analysis indicate that all samples exhibit 1T-MoS_2_ and that thiol molecules chemically bond to the MoS_2_ lattice. We therefore hypothesize that thiol groups are directly incorporated into and participate in the formation of the MoS_2_ layers. In other words, it can be assumed that thiols are acting as a sulfur source for MoS_2_ formation during the bottom-up hydrothermal synthesis. If all thiol groups of HDT participated in the MoS_2_ formation, the atomic ratio between S and Mo would remain constant at 2:1 across all samples. Therefore, we employed elemental analysis using both inductively coupled plasma optical emission spectroscopy (ICP-OES) and STEM-EDX (Figure 3C). While the S/Mo ratio is close to 2 for samples with low and medium HDT loadings (up to MoS_2_-HDT1), both methods independently indicate that the sulfur ratio is increasing significantly for high HDT-pillar loadings (MoS_2_-HDT2 and MoS_2_-HDT5). We consider the ICP-OES measurements to be quantitatively more reliable than EDX due to two primary factors: (1) the Mo Lα (∼2.3 keV) and S Kα (∼2.3 keV) peaks are very close in energy, complicating the spectral deconvolution, and (2) X-ray absorption and scattering effects related to sample thickness can distort the EDX quantification. The results lead to the conclusion that several sulfur bonding configurations are simultaneously present in pillared MoS_2_ samples, for which possible options are schematically depicted in Figure 3D. Sulfur in MoS_2_ not associated with an HDT molecule is labeled S1 (Mo-S) and sulfur in MoS_2_ directly associated with an HDT molecule is labeled S2 (Mo-S-C-) (“ideal MoS_2_”). Configurations responsible for S/Mo ratios greater than two (“non-ideal MoS_2_”) can be sulfur in MoS_2_ (S1-type) which additionally bonds with a thiol group, which is then labeled S3 (Mo-S-S-C-) or a sulfur from a thiol group (S2-type) bonding with a sulfur in MoS_2_ (S1-type), which is then labeled S3′ (Mo-S-S-Mo).

XPS is employed to investigate the proposed sulfur bonding configurations in pristine MoS_2_, as well as in pillared MoS_2_ with medium (MoS_2_-HDT1) and high (MoS_2_-HDT2) HDT-loading. In Figure 3E, the Mo 3d and S 2s spectra are presented. The Mo 3d region of pristine MoS_2_ is fitted with three doublets and two single peaks corresponding to the S 2s region at low binding energies. The three doublet peaks at 229.3, 230.2, and 233.0 eV correspond to Mo^4+^ from the ideal and “non-ideal” MoS_2_ (e.g., from the sulfur in the edges or poly sulfur species)^23^ and Mo^6+^, respectively. This result reveals the formation of 1T-MoS_2_, in agreement with the Raman spectrum (Figure 3B).^24^ The display of the Mo^6+^ doublet peaks indicates the presence of some surface oxidation and/or traces of unreacted MoO_3_ precursor. Meanwhile, the two doublets of Mo^4+^ suggested that the ideal stoichiometry of MoS_2_ is synthesized (i.e., S1), as well as other poly sulfurs, such as the formation of disulfides bonded chemically to Mo atoms, as indicated in Figure 3D (i.e., S3′). This assumption is in agreement with the S 2s and S 2p (Figure 3F) regions. The sulfur signal shows a main peak corresponding to S^2–^ from MoS_2_ (Mo-S1) and a less intense peak at higher binding energies attributed to the S–S bond (S3′, Mo-S-S-Mo).^23^ It should be noted that the presence of the second doublet of Mo^4+^ could alternatively be correlated with 2H-MoS_2_ instead of “non-ideal” MoS_2_ with disulfide formation.^25^ However, Raman results and the complete absence of any irreversible capacity during the initial electrochemical lithiation of the pristine MoS_2_ sample (discussion in the electrochemistry section, vide infra) strongly point toward exclusive 1T-MoS_2_ formation in our samples.

In both HDT-functionalized samples, the Mo^4+^ 3d_5/3_ peaks shift slightly to 229.1 eV (MoS_2_-HDT1) and 229.0 eV (MoS_2_-HDT2), indicating that molybdenum loses electrons to sulfur which can be explained by the formation of covalent bonds with the HDT pillars, in agreement with FTIR results. In addition, the intensity of the peak corresponding to disulfides is increasing, more significantly in the densely pillared MoS_2_-HDT2, suggesting the formation of more “non-ideal” Mo-S-S-Mo bonds. In line with the peak increase in S 2s, a new doublet peak is displayed in the S 2p region (Figure 3F). Considering that it appears at binding energies between Mo-S and Mo-S-S-Mo bonds, it could identify the new bond as Mo-S-C- (S2) or Mo-S-S-C- (S3) bonds. The ICP-OES and STEM-EDX analyses indicate the presence of a higher sulfur concentration in the MoS_2_-HDT2 sample, suggesting that S2-type bonds (Mo-S-C-) are more prevalent in less densely pillared MoS_2_, whereas S3-type bonds (Mo-S-S-C-) become more dominant at higher pillar densities. Although S3-type bonds are less stable compared to S2-type bonds, their formation might be facilitated under the higher HDT concentrations during the bottom-up synthesis.

Overall, the correlation between the XPS results and elemental composition (S/Mo ratio) from ICP-OES and STEM-EDX confirms the presence of various sulfur configurations proposed in Figure 3D. These configurations reflect different sulfur bonding environments influenced by the increasing loading of HDT in pillared MoS_2_. The emergence of S3 and/or S3′ suggests distinct structural configurations, although their binding energies are likely very similar. These configurations also explain why the S/Mo atomic ratio increases with higher pillar loading in the samples. The underlying principles governing the formation of these specific structures require further investigation by simulation, which is carried out in the following.

### Structure Optimization by Density Functional Theory Simulation

Atomistic models of pristine and HDT-pillared MoS_2_ are simulated via density functional theory (DFT) calculations (Figure 4A,B). The optimized interlayer spacing for pristine MoS_2_ is 0.601 nm, whereas HDT-inserted MoS_2_ with 25% pillar site occupancy exhibits an expanded interlayer spacing of 1.088 nm. These results predicted by DFT simulations align closely with the XRD patterns for pristine MoS_2_ and MoS_2_-HDT2 shown in Figure 1B, confirming the structural changes upon pillaring. Notably, the simulated HDT-pillared MoS_2_ model reveals a covalent connection between the MoS_2_ layers and HDT pillars, established by shared sulfur atoms. The lowest-energy configuration of alkyl chains between MoS_2_ layers occurs when the pillars adopt a zigzag arrangement (Figure S4). Additionally, DFT simulations of pristine MoS_2_ show that the interlayer distance follows a Lennard-Jones-like potential (Figure S5), suggesting that interlayer expansion results from the interplay between the energy cost of layer expansion and the stabilization energy gained from the adsorbed pillars.

**Figure 4:**
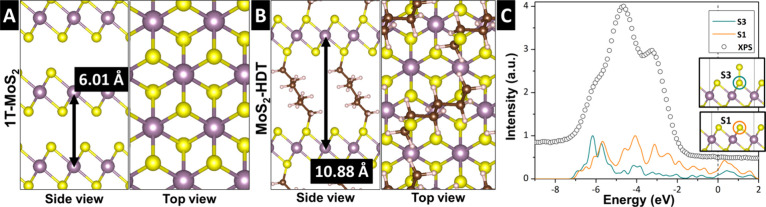
Simulated stable atomistic models of (A) pristine MoS_2_ and (B) functionalized MoS_2_-HDT_0.25_, with optimized interlayer *d*-spacing indicated by a black arrow. (C) The DOS for the sulfur p orbital in pristine MoS_2_ (orange) and p orbital of a sulfur atom in MoS_2_ with an extra bonded sulfur atom (blue), both found to be stable configurations, compared with the experimental XPS spectrum of MoS_2_-HDT2 taken from Figure 3F (circles). The dashed line represents the Fermi level of the simulated systems.

The insertion of HDT pillars promotes the formation of pillar domains, which minimizes energy loss associated with layer expansion at low pillar loading. This is in line with broadening of XRD (001) signals due to the low periodicity of the expanded MoS_2_ sheets. As these pillar domains grow and densify with higher pillar loading, the interlayer expansion becomes more homogeneous. This gradual domain growth explains abrupt evolution of the (001) ring in SAED and the significant peak shift in XRD observed between MoS_2_-HDT1 and MoS_2_-HDT2. The clustering of HDT pillars causes the broad distribution of the interlayer spacings, as observed in XRD and TEM/SAED. However, for high HDT-pillar loadings, the MoS_2_ layers show an increasingly homogeneous interlayer spacing. This dense arrangement may facilitate S–S bonding between the MoS_2_ layers and HDT pillars, particularly at the disordered layer edges.

To further investigate the relationship between pillar density and the formation of S–S bonds, *ab initio* electronic structure analysis of sulfur atoms is performed. The calculated density of states (DOS) of a sulfur atom in pristine MoS_2_ (S1) and a sulfur atom bonded to an additional sulfur atom (S3) have been compared with the experimental XPS spectrum (Figure 4C). We calculate an energy shift of approximately 3 eV upon S–S bond formation, which corresponds to the emergence of S3-type sulfur peaks in the XPS spectrum (Figure 3F). DOS analysis using a model that includes the HDT pillar reveals a similar energy shift in S3-type sulfur (Figure S6). Given the hydrothermal synthesis conditions and the high concentration of HDT molecules serving as a sulfur precursor, the formation of S–S bonds is plausible. The bonding likely occurs not only within the bulk material but also at the surface, defects, and edges within disordered pillar domains.

### Electrochemical Characterization

The electrochemical properties of pristine and HDT-pillared MoS_2_ are evaluated as host electrode materials for Li^+^ intercalation in standard organic electrolyte (LP30). Given the comparable morphology and 1T phase of all samples, we can unambiguously establish a connection between the electrochemistry and the specific nanoconfinement properties of interlayer-expanded, covalent networks of HDT-pillared MoS_2_ and analyze the impact of pillar loading on ion transport and storage in detail. To exclude the influence of electrolyte wetting effects in electrodes with varying pillar loading, we have also monitored the open circuit voltage over 12 h before starting the measurements,^26^ ensuring stabilization of the open circuit voltage (OCV) prior to measurement (Figure S7).

Cyclic voltammograms (CV) of the first (de)­lithiation cycle are shown for all samples (Figure 5A). While pristine MoS_2_ exhibits a quasi-rectangular CV typical for pseudocapacitive lithium intercalation,^8,27–29^ a reduction peak can be seen for all HDT-pillared samples that increases in intensity and shifts toward lower potentials for increasing HDT-loading from MoS_2_-HDT0.2 to MoS_2_-HDT2. In contrast, the subsequent oxidation peak is located at around 2.2 V for all these samples. The CV of MoS_2_-HDT5 shows a drastic reduction in charge storage capacity. Interestingly, the magnitude of the redox peaks appears linked to the pillar loading, with increasing peak currents going from pristine MoS_2_ to MoS_2_-HDT1 (before rapidly decreasing for high pillar loadings). The findings indicate that covalent pillaring leads to stronger ion-host interaction manifested in an increased redox signature.^30^ This is in contrast to noncovalent pillaring, for example, with hexanediamine pillars (HDA) that we have investigated previously.^8^ While the addition of noncovalently interacting HDA to MoS_2_ led to an increasingly rectangular/pseudocapacitive CV signature, covalently interacting HDT yields the opposite effect. This underlines the crucial importance of host-pillar interaction on the resulting electrochemical response.

**Figure 5:**
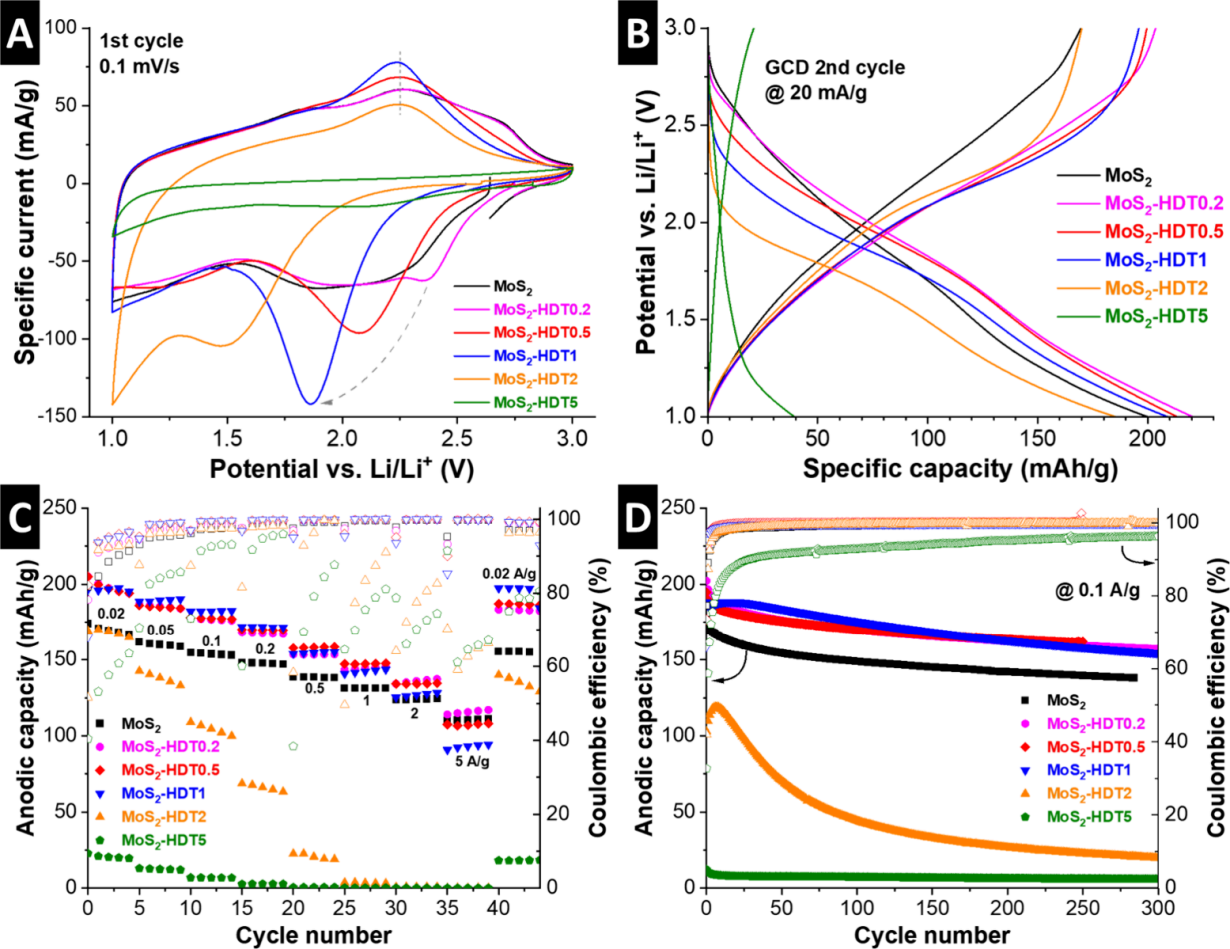
Electrochemical performance of MoS_2_ and HDT-functionalized MoS_2_. Comparison of (A) CVs at 0.1 mV/s (first cycle) and (B) GCD curves at 20 mA/g (second cycle). (C) Rate handling at specific currents of 0.02, 0.05, 0.1, 0.2, 0.5, 1, 2, and 5 A/g. (D) Galvanostatic cycling stability at a constant current of 0.1 A/g. All measurements are carried out in coin cells versus Li metal with LP30 electrolyte at a constant temperature of 20 °C.

In subsequent CV cycles (Figures S8, S9), the reduction peak converges around 1.7–1.8 V for all pillared MoS_2_ samples with different loadings (MoS_2_-HDT0.2 to MoS_2_-HDT2). We hypothesize that the initial reduction peak at varying potentials is due to an activation process involving rearrangement of HDT pillars during the first lithiation cycle that is necessary to facilitate favorable Li^+^ diffusion pathways. Impedance spectroscopy and DFT analysis will further support this hypothesis, vide infra.

Galvanostatic charge/discharge (GCD) is employed to examine the maximum charge storage capacity of the samples during initial cycles at a low specific current of 20 mA/g (Figure 5B, Figure S10). The specific delithiation capacities of HDT-pillared MoS_2_ with low to medium loading are in a comparable range with 196, 200, and 204 mAh/g for MoS_2_-HDT1, MoS_2_-HDT0.5 and MoS_2_-HDT0.2, respectively, and it is 170 mAh/g for both pristine MoS_2_ and MoS_2_-HDT2. When taking into account the increasing mass contribution of HDT pillars, the capacity per MoS_2_ increases up to medium pillar loading. The maximum reversible (de)­lithiation capacities for the samples are Li_1.01_MoS_2_, Li_1.33_MoS_2_-HDT0.2, Li_1.36_MoS_2_-HDT0.5, Li_1.43_MoS2-HDT1, and Li_1.40_MoS2-HDT2. MoS_2_-HDT5 with the highest pillar loading exhibits highly reduced capacity (21 mAh/g or Li_0.25_MoS_2_-HDT5), explainable by blocking of active sites by excessive pillar loading, as demonstrated by DFT vide infra.

The rate handling behavior of the samples is further quantified by GCD at various specific currents from 0.02–5 A/g (Figure 5C). At increased rates, the samples with the lowest pillar loading showed superior capacity retention, which is similar to pristine MoS_2_. The highest performance is exhibited by the sample with the lowest pillar loading, MoS_2_-HDT0.2, with a retention of ca. 120 mAh/g at 5 A/g. For MoS_2_-HDT2 and MoS_2_-HDT5, the capacity drastically dropped at higher rates, indicative of poor kinetics caused by the high HDT-pillar loading.

Overall, the basic electrochemical characterization reveals the ambivalent influence of HDT-pillaring on the capacity and rate performance of the lithium intercalation reaction in MoS_2_. One the one hand, additional storage sites become activated in the interlayer galleries, even in the inhomogeneously expanded samples with low HDT loading. The capacity becomes maximized in samples with medium HDT loading, with homogeneously expanded interlayers, but before overcrowding with HDT takes place. The rate behavior benefits from low HDT loading, even if the interlayer expansion is inhomogeneous. This is due to the hindrance toward diffusion posed by HDT pillars, as is also shown by simulation, vide infra.

The cycling stability is tested by GCD at a medium rate of 0.1 A/g (Figure 5D). For samples up to a medium pillar loading, the stability after 200 cycles is positively impacted by a higher HDT content. The MoS_2_-HDT1 sample retains ca. 87% of its capacity after several hundreds of cycles, surpassing pristine MoS_2_ (80%), MoS_2_-HDT0.2 (80%), and MoS_2_-HDT0.5 (84%). These results indicate improved stability of the electrochemical (de)­lithiation process in covalently pillared MoS_2_ materials. Samples with high pillar loading, however, rapidly fade in capacity.

The impact of HDT pillar content on the (de)­lithiation kinetics is studied in detail using electrochemical impedance spectroscopy (EIS) at various states of charge over the full potential window of 3.0 – 1.0 V vs Li/Li^+^ during the first lithiation. We focus the analysis on understanding the reduction peak in the first cycle, which we explained by an “activation”/pillar rearrangement process during initial lithiation. Therefore, impedance spectra of individual electrodes are shown at 2.6 V vs Li/Li^+^ (roughly the OCV for each electrode), at the onset potential of the reduction process of each individual electrode and in the (almost) fully lithiated state at 1.2 V vs Li/Li^+^ (Figure 6A–C). For pristine MoS_2_, the Nyquist plots show comparable shape and magnitude at each of the probed potentials, with a pronounced charge transfer resistance in the midfrequency region and comparable open Warburg-type behavior in the low-frequency region indicative of finite-length diffusion (Figure 6A).

**Figure 6:**
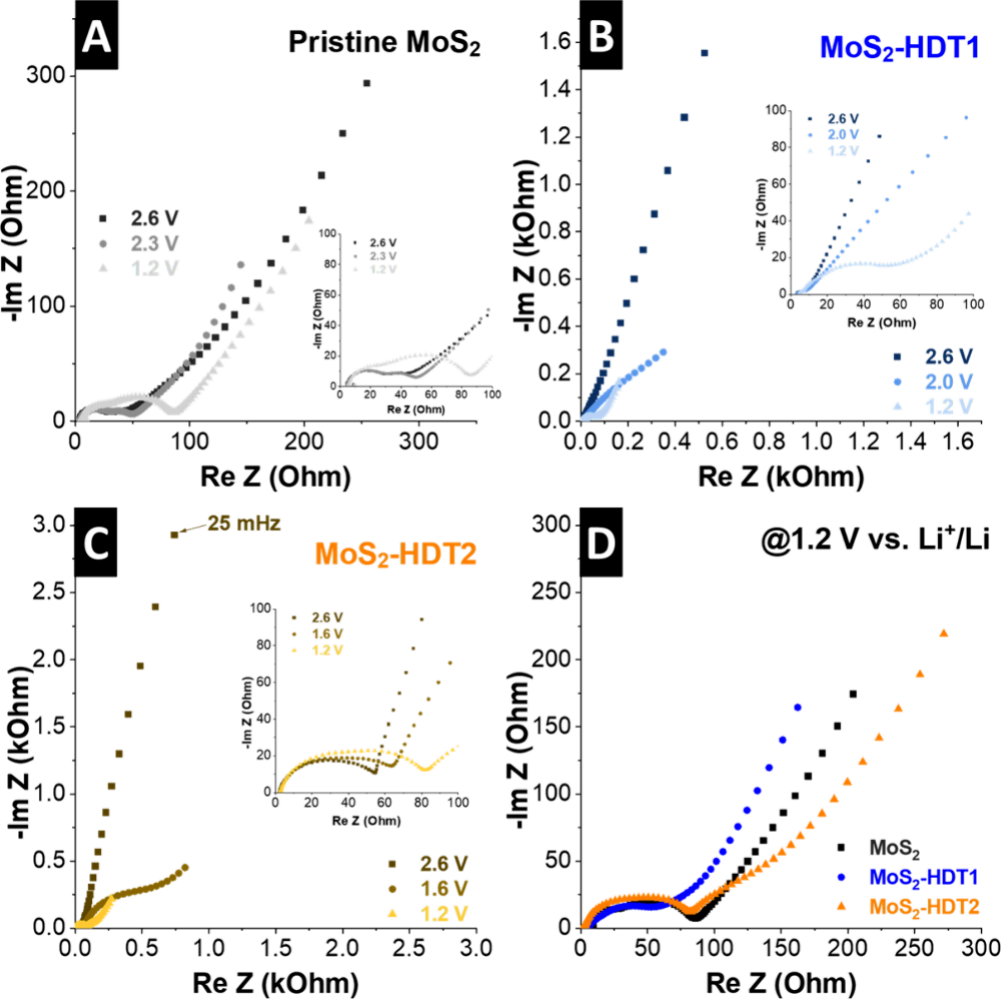
Nyquist plots at various electrode potentials versus Li/Li^+^ of (A) pristine MoS_2_, (B) MoS_2_-HDT1, and (C) MoS_2_-HDT2. (D) Comparison of lithiated electrodes at 1.2 V vs Li/Li^+^.

For pillared MoS_2_ electrodes with medium and high pillar density, a drastic change in the Nyquist plots is observed as a function of electrode potential. At OCV, both electrodes show blocking electrode behavior with large bending arcs in the low frequency region (Figure 6B,C). Moreover, both electrodes show a transition in the Nyquist plot shape around the potential region where their initial reduction peak occurs (Figure 5A), which is around 2.0 V for MoS_2_-HDT1 and around 1.6 V for MoS_2_-HDT2. The results indicate that the interlayer spaces of pillared MoS_2_ electrodes only become accessible for Li^+^ intercalation after the initial, irreversible reduction process. It is noteworthy that stronger reductive potentials are required with increasing HDT pillar densities. Finally, in their lithiated state (1.2 V), all three electrodes show comparable charge transfer resistance in the midfrequency region, while impedance in the Warburg-region associated with ion diffusion is most pronounced for MoS_2_-HDT2 (Figure 6D). This confirms the impeded ion transport in crowded interlayer space with high pillar loading. It should be noted that all electrodes exhibit an increased charge transfer resistance in the midfrequency range in their lithiated states, which can be explained by the formation of an interfacial layer at highly reductive potentials.^31^ We also fitted the EIS data with an equivalent circuit model, as indicated in Figure S11. The mid-frequency region consists of two semicircles, which indicate the presence of a surface film and a charge transfer resistance after initial reduction.^32^

### Atomistic Simulation of Electrochemical Lithiation Process

To elucidate the significant differences in maximum lithiation capacity and solid-state diffusion of Li^+^ as a function of pillar density, lithiated structures of pristine and HDT-pillared MoS_2_ are compared using DFT optimization (Figure 7) and climbing image nudged elastic band (CI-NEB) via DFT (Figure 8). Simulations indicate that an increased interlayer distance can accommodate twice the number of Li^+^ compared to the pristine material (Figure 7A) by providing additional space at the Mo top sites above and below within the interlayer (Figure 7B). However, while the pillars are essential for increasing the interlayer distance, they also hinder lithiation by blocking potential Li^+^ sites, resulting in a charge capacity that is less than the anticipated doubled increase (Figure 5D and Figure S10). The increased lithiation sites contribute to the enhanced capacity observed in MoS_2_-HDT samples with moderate pillar density. In contrast, in systems with high pillar density, such as MoS_2_-HDT5, most lithiation sites are occupied by pillars, significantly reducing capacity. The high density of pillars occupying potential lithiation sites results in decreased charge storage capacity. The change in the optimized interlayer distance before and after full lithiation in pristine MoS_2_ (+9%) is moderate but significantly higher than that of MoS_2_-HDT_0.25_ (+1%) (Figure 7). This arises from the already extended interlayer distance in MoS_2_-HDT_0.25_ that is less susceptible to volumetric expansion upon lithiation compared to pristine MoS_2_.

**Figure 7:**
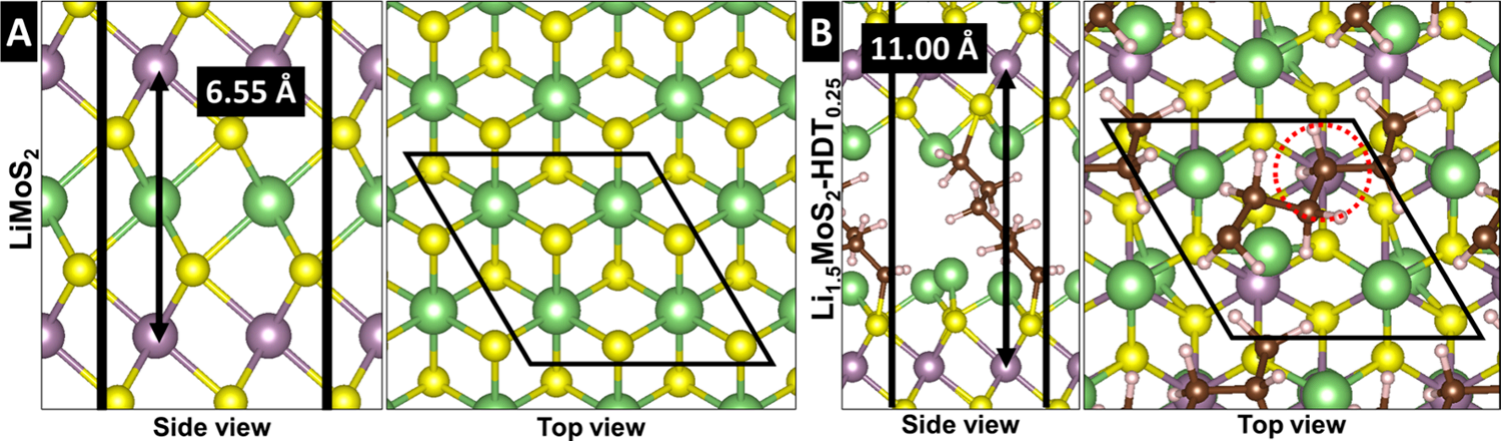
Simulated stable atomistic models of fully lithiated (A) Li_1.0_MoS_2_ and (B) Li_1.5_MoS_2_-HDT_0.25_. Green spheres represent Li atoms. The optimized *d*-spacing with Li atoms is indicated with a black arrow. The black box outlines the simulated cell under the periodic boundary condition. Lithiation at the potential Li site (red circle) is inhibited by the pillar.

**Figure 8:**
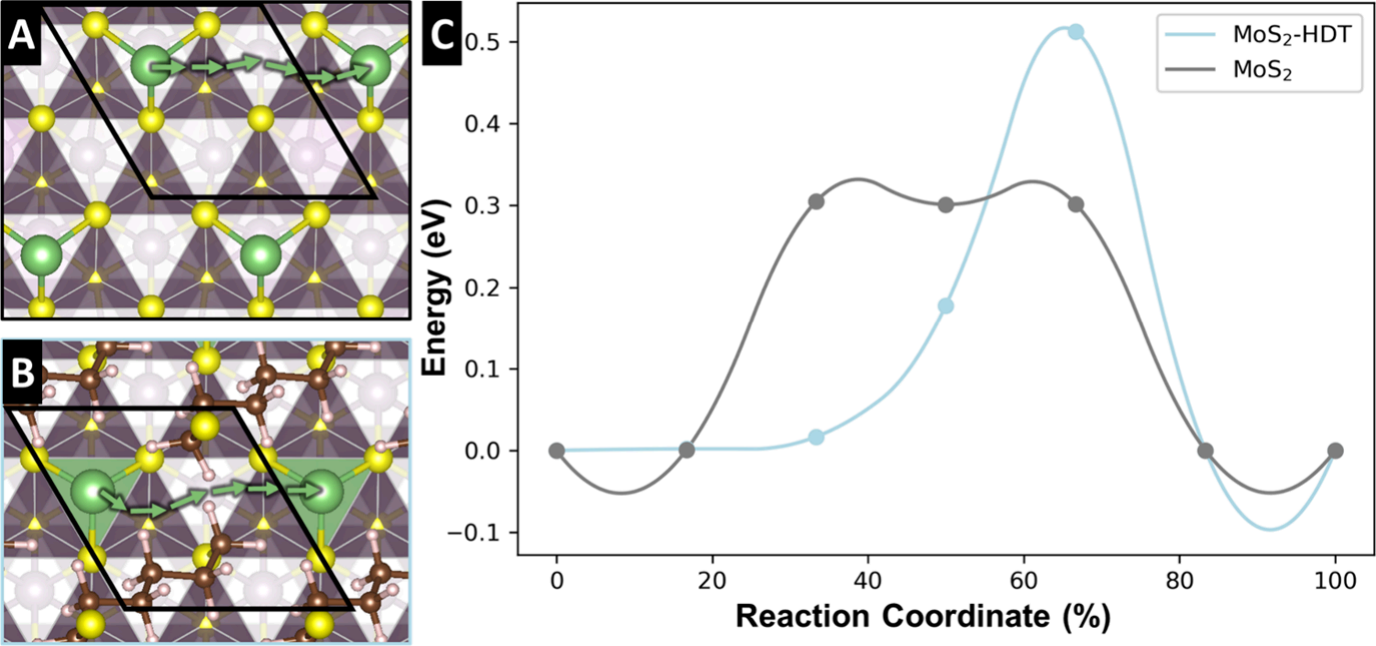
Top view of the Li^+^ ion diffusion pathways in (A) MoS_2_ and (B) MoS_2_-HDT. (C) The energy profiles are plotted against the reaction coordinates. Green arrows in (A) and (B) represent the Li^+^ diffusion pathway at each data point from (C). The interlayer distances are derived from Figure 4A and Figure 4B, respectively. The black box outlines the simulated cell under the periodic boundary condition. The complete images from pathways A and B are available in Figure S12.

The energy barrier for Li^+^ diffusion pathways is simulated for both pristine MoS_2_ and MoS_2_-HDT_0.25_ (Figure 8). The Li^+^ diffusion barrier is 0.30 eV for MoS_2_ and 0.51 eV for MoS_2_-HDT_0.25_. In pristine MoS_2_, the Li^+^ ion diffuses along sulfur atoms with a low energy barrier (Figure 8A). However, in HDT-pillared MoS_2_, the pillars impede Li^+^ ion diffusion, increasing the energy barrier (Figure 8B). The differences in energy barriers between the two systems suggest that Li^+^ diffusion preferably occurs outside or around the perimeter of the pillar-inserted domains. The restructuring of the inserted pillars, driven by charge carrier diffusion affects the stability of the inserted pillar, also serves as an explanation for poor capacity retention of pillared MoS_2_ with high pillar loadings. Hence our calculations emphasize the importance of balancing the pillar loading within covalently pillared MoS_2_ host materials. While low HDT pillar loadings improve the lithiation capacity, increasing HDT loadings leads to active site occupation and, crucially, impeded Li^+^ transport within the interlayer space.

## Conclusions

In this work, we have demonstrated a strategy for tailoring the nanoconfinement environment in MoS_2_ intercalation host materials via pillaring with 1,6-hexanedithiol molecules. The materials consist of covalent networks between HDT pillars and the MoS_2_ lattice and we were able to demonstrate a systematic variation in HDT pillar loading. Structural analyses by XRD, TEM, and SAED revealed that, at low to medium pillar loadings, the interlayer expansion is irregular because of the formation of thermodynamically preferential HDT domains, whereas high HDT loadings promote a more homogeneous expansion of MoS_2_. Combination of XPS, elemental analysis and DFT calculations reveal the presence of several sulfur configurations depending on the HDT loading. While at low to medium loading, most pillars are directly incorporated in the lattice forming “ideal MoS_2_” (Mo-S-C), high pillar loadings lead to increased formation of “non-ideal MoS_2_” (Mo-S-S-C and Mo-S-S-Mo). Hence, we developed a comprehensive understanding of the structure of covalently pillared MoS_2_ materials.

Electrochemical characterization reveals an increased storage capacity in pillared MoS_2_. DFT simulation demonstrated that this is due to the availability of additional storage sites in the expanded interlayer, and a maximum reversible storage capacity of 1.43 Li^+^ was measured experimentally in MoS_2_-HDT1. However, it was shown that HDT pillars occupy Li^+^ storage sites, hence at high pillar loadings, the capacity rapidly diminishes. Excessive pillar densities impede Li^+^ diffusion by increasing the energy barrier along diffusion paths.

Overall, the study provides a comprehensive understanding of the structure and covalent interaction in HDT-pillared MoS_2_ as a function of HDT loading and the resulting lithium intercalation capacity and kinetics. It presents covalent pillaring with thiols as a promising strategy for transition metal dichalcogenides used as ion intercalation hosts materials. Crucially, the impact of pillar loading on the electrochemical performance is emphasized, underlining the importance of controlling materials structure to guide functional properties.

## Methods

### Materials Synthesis

#### Synthesis of Pristine MoS_2_

Molybdenum disulfide (MoS_2_) was synthesized via a one-pot hydrothermal method. In a typical procedure, 150 mg of MoO_3_ (Alfa Aesar), 175 mg of thioacetamide (Thermo Fisher Scientific), and 1.5 g of urea (Merck KGaA) were dissolved in 25 mL of deionized (DI) water and stirred for 30 min in a glass beaker. The pH of the hydrothermal solution was adjusted to approximately 2 by adding diluted hydrochloric acid (HCl), as monitored with a Blueline 14 pH meter (Xylem Analytics). The resulting solution was transferred into a 100 mL Teflon vessel within an autoclave (BP-100 high-pressure reactor, Berghof). The synthesis was conducted at 180 °C for 12 h, with heating and cooling periods of 2 h each. After synthesis, the reaction products were washed with DI water and ethanol via vacuum filtration. The material retained on a 0.22 μm hydrophilic PTFE membrane (Millipore) was collected by rinsing with 15–20 mL of DI water into a 50 mL centrifuge tube. The resulting MoS_2_ slurry was dispersed by shaking and sonication for approximately 3 min. The well-dispersed solution was then frozen in liquid nitrogen, and the frozen sample was transferred directly to a freeze-dryer (Alpha 3-4 LSCbasic, Martin Christ) for 72 h. The resulting fine black MoS_2_ powder was collected for further use.

#### Synthesis of HDT-Functionalized MoS_2_

Functionalized MoS_2_ with 1,6-hexanedithiol (HDT) (Thermo Fisher Scientific) was synthesized following the same procedure as for pristine MoS_2_, with two additional steps. First, HDT was added to the 25 mL precursor solution in molar ratios of 0.2, 0.5, 1, 2, and 5 relative to molybdenum (Mo). Second, after the hydrothermal reaction, the products were washed with acetone during vacuum filtration to remove any unreacted HDT molecules. All collected powders were then dried in an 80 °C oven for 20–30 min prior to characterization.

### Materials Characterization

#### Powder X-ray Diffraction (XRD)

XRD patterns were recorded in Bragg–Brentano geometry using a Bruker D8 Advance diffractometer equipped with a Cu Kα radiation source (λ = 1.5406 Å), with a step size of 0.02° and a dwell time of 1 s.

#### Thermogravimetric Analysis (TGA)

TGA was conducted using a Discovery TGA 7 (TA Instruments) under a 1:1 oxygen/nitrogen gas flow (20 mL/min) at a heating rate of 2 K/min.

#### Nitrogen Sorption

Samples were degassed at 120 °C for 12 h prior to measurement. Nitrogen (N_2_) adsorption–desorption isotherms were recorded using 34 data points for both adsorption and desorption branches. The Brunauer–Emmett–Teller (BET) surface area^19^ of each sample was calculated using the multipoint BET method based on five selected points in the linear region of the isotherm.

#### Raman Spectroscopy

Raman spectra were acquired using a Renishaw InVia confocal Raman microscope with a 532 nm excitation laser. The laser power was maintained at 0.5 mW, and at least three different spots were analyzed to assess sample homogeneity.

#### Fourier Transform Infrared Spectroscopy (FTIR)

FTIR measurements were performed using a Spectrum Two FTIR spectrometer (PerkinElmer) at resolutions of 8 and 16 cm^–1^ over 20 scans, covering the spectral range from 400 to 4000 cm^–1^.

#### Microscopy

The morphology of the MoS_2_-based materials was examined by scanning electron microscopy (SEM) using a field emission SEM (Crossbeam x340, Zeiss) operated at 5 kV. Transmission electron microscopy (TEM) was performed on a Talos F200i (Thermo Fisher Scientific) at an accelerating voltage of 80 kV. For TEM sample preparation, the powders were gently ground with a pestle and mortar, and a carbon-coated Formvar film on a copper TEM grid was carefully rubbed onto the fine powder. Energy-dispersive X-ray spectroscopy (EDX) was carried out using Bruker DualX windowless detectors in scanning TEM (STEM) mode with a high-angle annular dark-field (HAADF) detector to provide spatially resolved elemental composition.

#### Elemental Analysis

The sulfur (S) to molybdenum (Mo) ratio was determined using an inductively coupled plasma–optical emission spectrometer (ICP-OES, SPECTRO ARCOS, AMETEK). The instrument was initially calibrated using 1000 mg/L ICP standard solutions for S and Mo (Merck). Approximately 20–35 mg of MoS_2_-based powder was digested in 4 mL of aqua regia using a Mars 6 microwave digestion system (CEM) with One-Touch technology.

#### X-ray Photoelectron Spectroscopy (XPS)

XPS measurements were conducted in a SPECS UHV system (FOCUS 500) equipped with a monochromatic Al Kα source (hν = 1486.6 eV) and a PHOIBOS 150 hemispherical analyzer with a 2D DLD detector (Surface Concept). High-resolution spectra were collected at 200 W (12 kV) with a pass energy of 30 eV and a step size of 0.1 eV. Spectral fitting was performed using CasaXPS software (employing a Shirley background and GL(30) line shape), with calibration based on the C–C/C–H sp^3^ signal at 284.8 eV.^33^

### Electrode Preparation and Electrochemical Characterization

#### Electrode Fabrication

A slurry was prepared by mixing the active material (pristine MoS_2_ or HDT-functionalized MoS_2_), carbon black (CB), and polyvinylidene difluoride (PVDF) in a weight ratio of 8:1:1. Initially, the MoS_2_-based powders and CB were ground together using a pestle and mortar. The mixture was then transferred to a small container, and a 2 wt % PVDF solution in *N*-methyl-2-pyrrolidone (NMP) was added. The slurry was homogenized using a speed mixer (ARE-250, Thinky) at 1000 rpm for 10 min. The resulting slurry was coated onto carbon-coated aluminum foil using a doctor blade (wet film thickness of 90 μm) and dried overnight at 80 °C to remove the NMP solvent. Circular electrodes (12 mm diameter) were punched from the dried film and further dried overnight at 80 °C before transfer to an argon-filled glovebox. The mass loading of MoS_2_ of electrodes is between 0.9 and 1.2 mg/cm^2^. Mass normalizations refer to the 80 wt % of active material within the electrodes, omitting the mass of inactive PVDF binder and CB.

#### Cell Assembly and Electrochemical Testing

Metallic lithium discs (14 mm, Honjo) served as the negative electrodes. 2032-type coin cells (Hohsen) were assembled inside a glovebox (MBraun; O_2_, H_2_O < 0.5 ppm), incorporating a 1 mm thick stainless steel spacer, a stainless steel spring, and a glass microfiber separator (Whatman grade GF/A). Each cell was filled with 120 μL of LP30 electrolyte (1 M LiPF_6_ in a 1:1 volumetric mixture of ethylene carbonate (EC) and dimethyl carbonate (DMC), Solvionic). Cyclic voltammetry (CV) and galvanostatic charge/discharge (GCD) tests were performed using potentiostats (Bio-Logic VMP 3-e and VMP300) within a potential window of 1.0 to 3.0 V versus Li/Li^+^ at 20 °C (Binder temperature-controlled chambers). Long-term cycling tests were conducted at a current density of 0.1 A/g.

#### Electrochemical Impedance Spectroscopy (EIS)

EIS and in situ EIS measurements were performed using a three-electrode setup. For the three-electrode cell (T-cell, Swagelok), the working electrode (MoS_2_-based material on Al foil), the counter electrode (Li), and a glass microfiber separator (Whatman grade GF/D, 12 mm diameter) were assembled. A lithium reference electrode was positioned centrally in the T-cell, separated from the other electrodes by an 8 mm GF/D membrane. Initially, 120 μL of LP30 electrolyte was added between the working and counter electrodes, followed by an additional 120 μL beneath the reference electrode. The staircase potentio-electrochemical impedance spectroscopy (SPEIS) technique was used for in situ analysis at various states of charge. A total of 21 EIS spectra were collected during each cathodic and anodic cycle over a potential range of 3.0 to 1.0 V (vs Li/Li^+^), with a frequency range from 1 MHz to 10 mHz. Each frequency point was averaged three times, increasing logarithmically.

### Computational Details

Density functional theory (DFT) calculations were performed using QUANTUM ESPRESSO with orthogonal norm-conserving Vanderbilt (ONCV) pseudopotentials, utilizing the Perdew–Burke–Ernzerhof (PBE) exchange-correlation functional from the PseudoDojo library.^34–36^ For long-range dispersion corrections, the semiempirical van der Waals (vdW) correction by Grimme with zero damping (PBE+D3) was applied consistently throughout all calculations.^37^ The convergence criteria for total energy and forces were set at 10^–6^ Ry and 10^–4^ Ry bohr^–1^ or better, respectively. The Brillouin zone was integrated using a uniform reciprocal distance of 0.05 Å^–1^, resulting in Γ-centered (4 × 4 × 2) k-point grids for the bulk MoS_2_ (2 × 2 × 2) supercell. To simulate various *d*-spacings in MoS_2_, each MoS_2_ sheet was fixed with varied interlayer distances. For electronic structure calculations, self-consistent calculations were performed with twice denser k-point grids in the irreducible Brillouin zone. The climbing image nudged elastic band (CI-NEB) combined with dynamic nudged elastic band (dyNEB) method was employed to investigate Li^+^ mobility within the system.^38,39^ A (2 × 2 × 2) supercell was used for all calculations throughout this study. For the Li^+^ mobility study, simulations were converged better than f max <0.08 eV/Å.

## Supplementary Material



## Data Availability

Experimental data used in this work are made available on the Zenodo repository (https://zenodo.org) at https://doi.org/10.5281/zenodo.16872366. Simulation data used in this work are made available on Edmond (https://edmond.mpg.de/) at https://doi.org/10.17617/3.OWQRSO.

## Notes

Additional structural characterization (nitrogen sorption, scanning electron microscopy, elemental mapping), supporting simulation, and supporting electrochemical data (OCV, CVs, GCD, impedance) (PDF)
