# Supplementary material for: Interlayer Spacing Control of MoS2 with Covalent Thiol Functionalization: Understanding Structure and Electrochemistry from Experiments and Simulation
Source: ACS Nano. 2025 Oct 2;19(40):35425–37. doi: 10.1021/acsnano.5c07717 (PMC12539599; doi:10.1021/acsnano.5c07717)
Supplement: Supplementary file 1 [file nn5c07717_si_001.pdf]

Supporting Information

Interlayer spacing control of MoS_2 with covalent thiol functionalization: Understanding structure and electrochemistry from experiments and simulation

Jaehoon Choi,[1,2,+] Kyeonghyeon Nam,[3,+] Yoga T. Malik,[1,2] Robert Leiter,[1,2] Maider Zarrabeitia,[1,2] Christoph Scheurer,[3,4] Simon Fleischmann[1,2,*]

[1] Helmholtz Institute Ulm (HIU), Helmholtzstr. 11, 89081 Ulm, Germany

[2] Karlsruhe Institute of Technology (KIT), 76021 Karlsruhe, Germany

[3] Fritz Haber Institute of the Max Plank Society, 14195 Berlin, Germany

[4] IET-1, Forschungszentrum Jülich, 52425 Jülich, Germany

[*] Corresponding author's email: simon.fleischmann@kit.edu

[+] These authors contributed equally

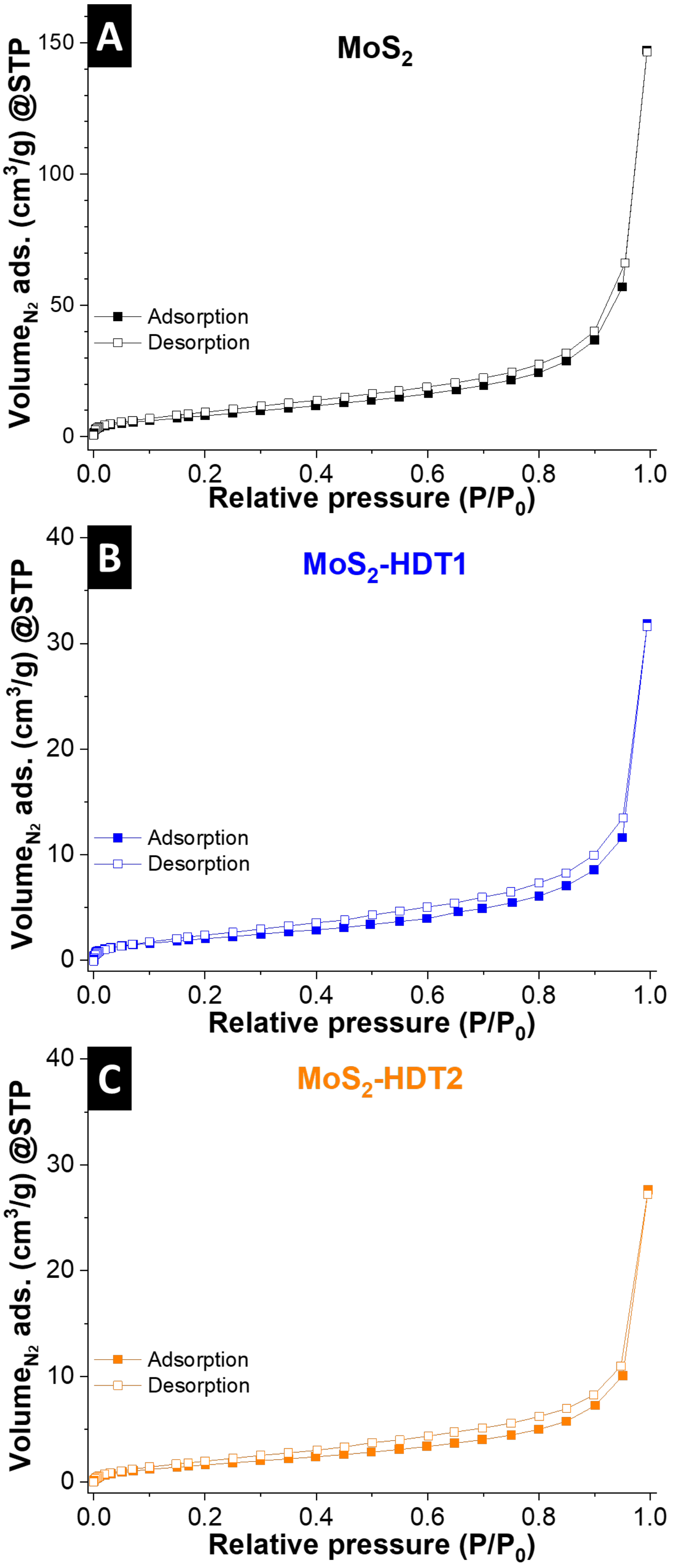

Fig. S1: Nitrogen adsorption-desorption isotherms of (a) pristine MoS_2, (b) MoS_2-HDT1, and (c) MoS_2-HDT2 samples.

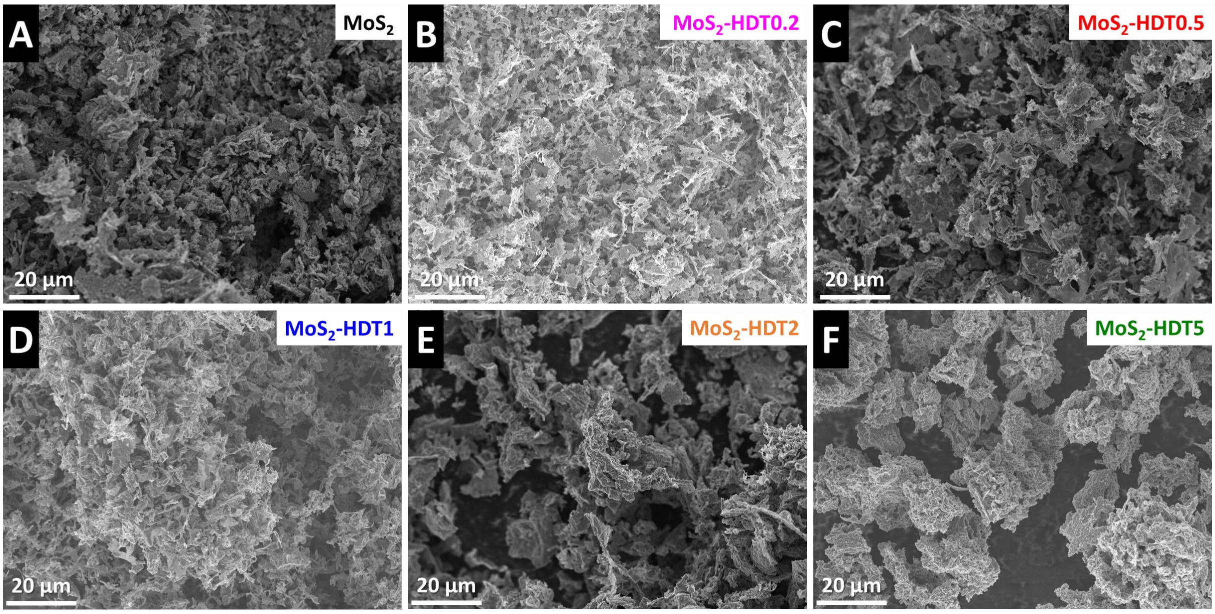

Fig. S2: Scanning electron micrographs of (A) MoS_2, (B) MoS_2-HDT0.2, (C) MoS_2-HDT0.5, (D) MoS_2-HDT1, (E) MoS_2-HDT2, and (F) MoS_2-HDT5.

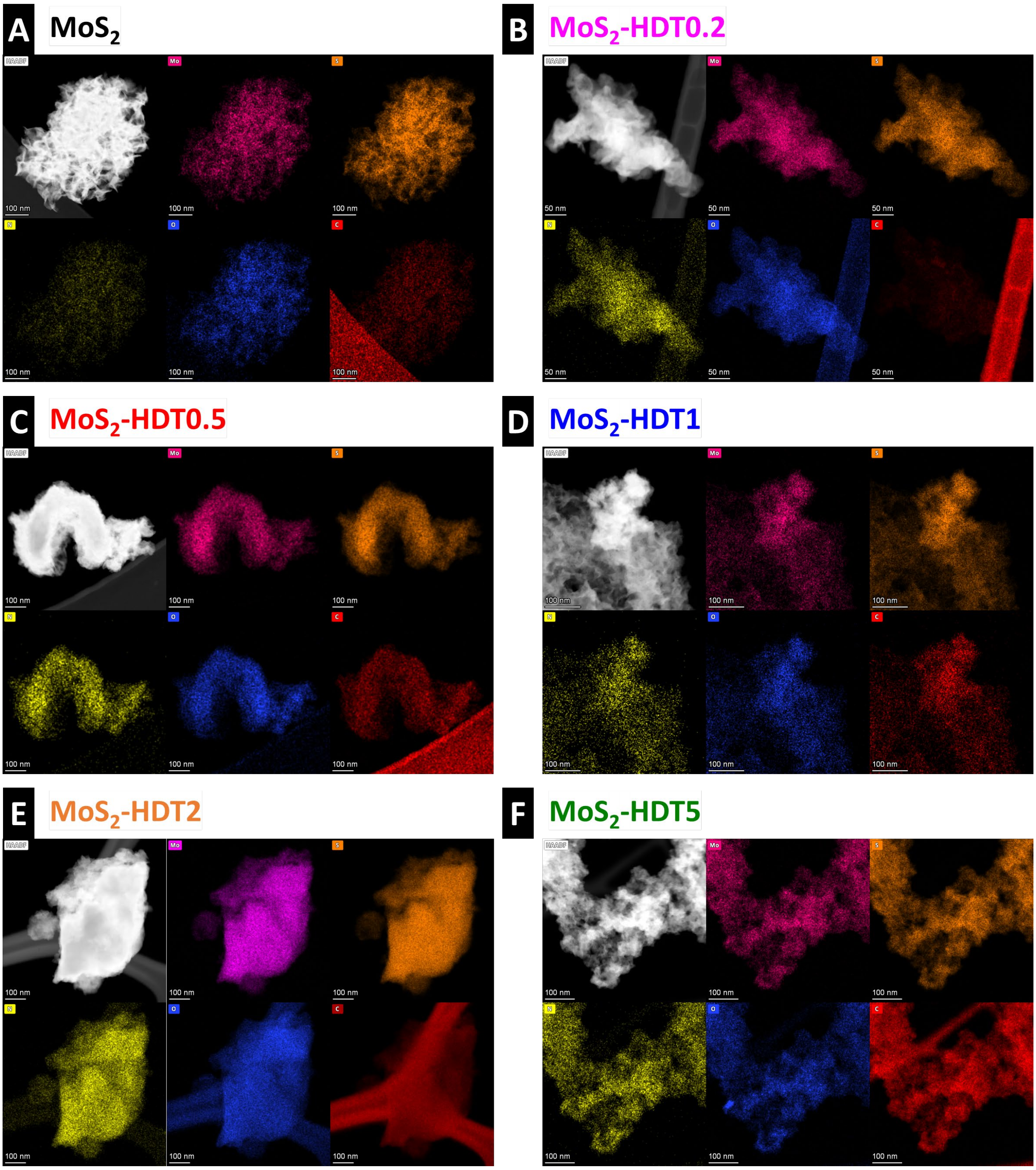

Fig. S3: Elemental mapping of molybdenum (pink), sulfur (orange), nitrogen (yellow), oxygen (blue), and carbon (red) by energy-dispersive X-ray spectroscopy in STEM mode. Inset: High-angle annular dark field images.

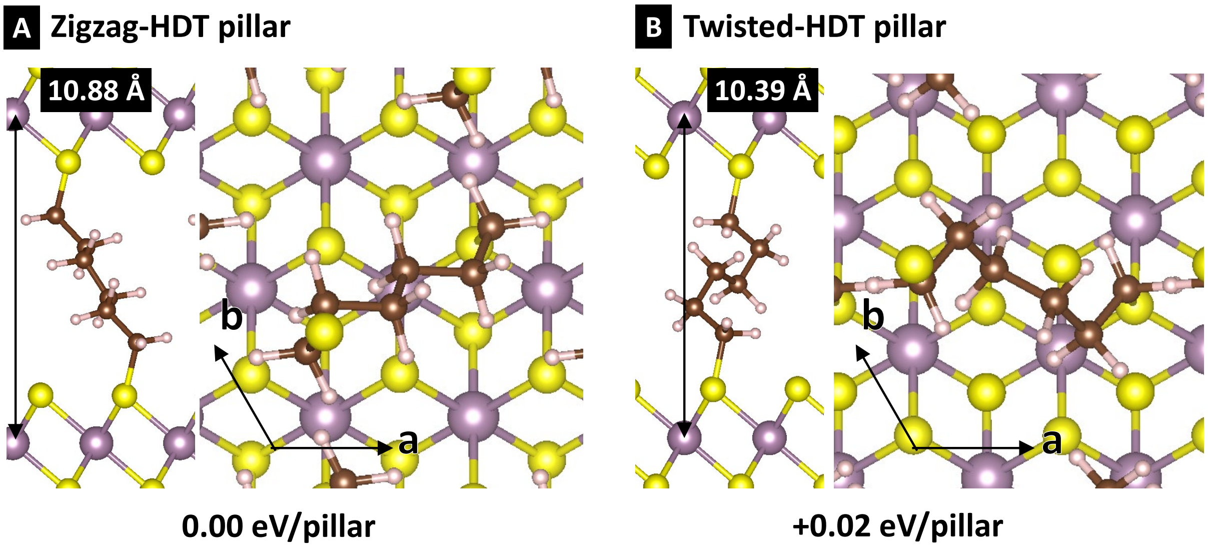

Fig. S4: The two most stable geometries of the inserted HDT pillar in MoS_2 were simulated and compared for both the "zigzag" and "twisted" structures as shown in side views (left) and top view (right). The relative energy is provided in eV per number of inserted pillars, as shown below.

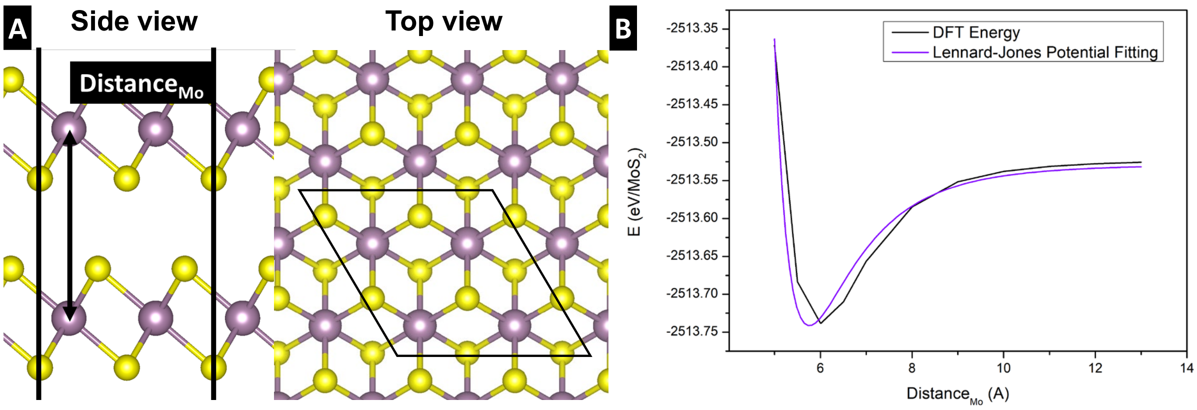

Fig. S5: The increase in Mo-Mo distance in pristine MoS_2 and the corresponding energy calculated using DFT were compared. The results follow the Lennard-Jones potential.

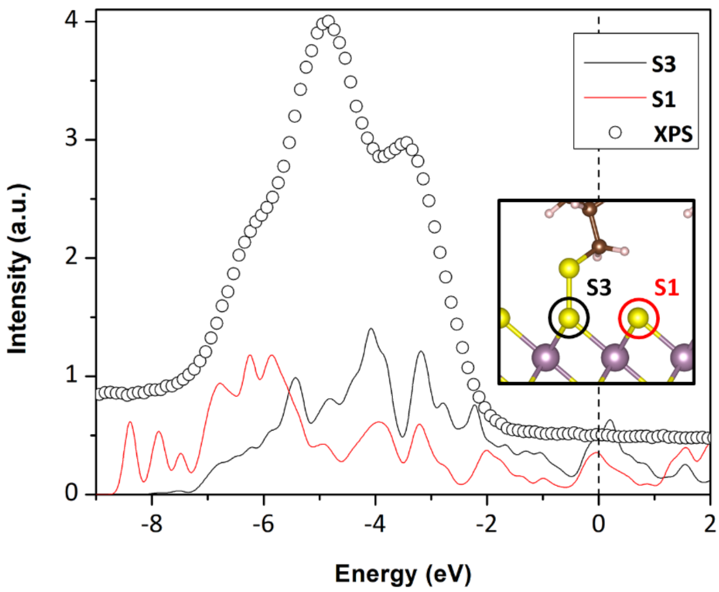

Fig. S6: The DOS for the sulfur p orbital in pristine MoS_2 (red) and p orbital of a sulfur atom in MoS_2 with an extra bonded HDT molecule (black), both found to be stable configurations, compared with the experimental XPS spectrum of MoS_2-HDT2 taken from **Fig. 3F** (circles).

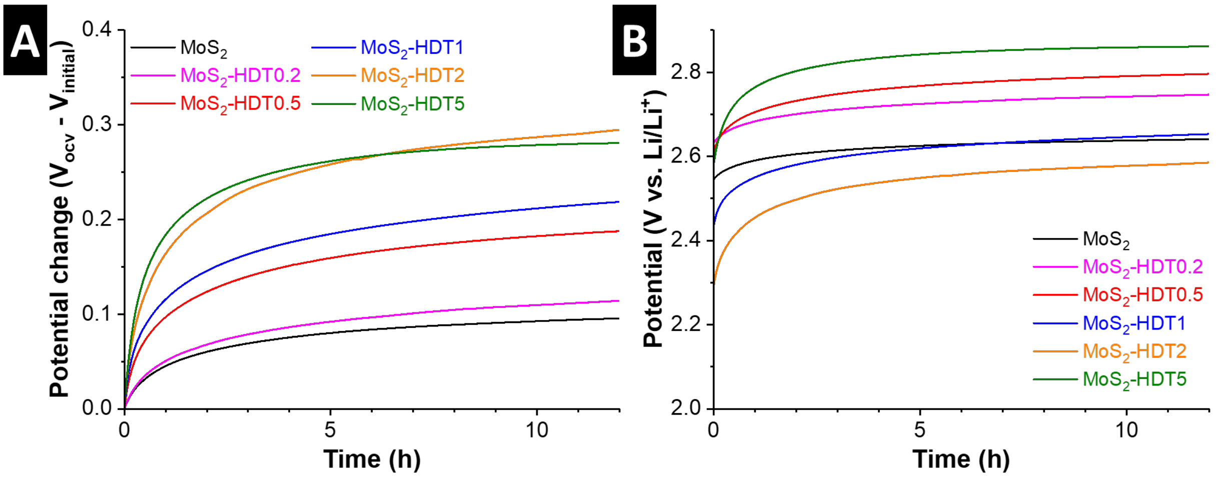

Fig. S7: Development of the open circuit voltage (OCV) of freshly assembled coin cells over 12 hours, prior to the start of the electrochemical measurements, (A) in terms of potential change over time, and (B) overall development of potential versus Li^+/Li, i.e., the cell voltage in the coin cell.

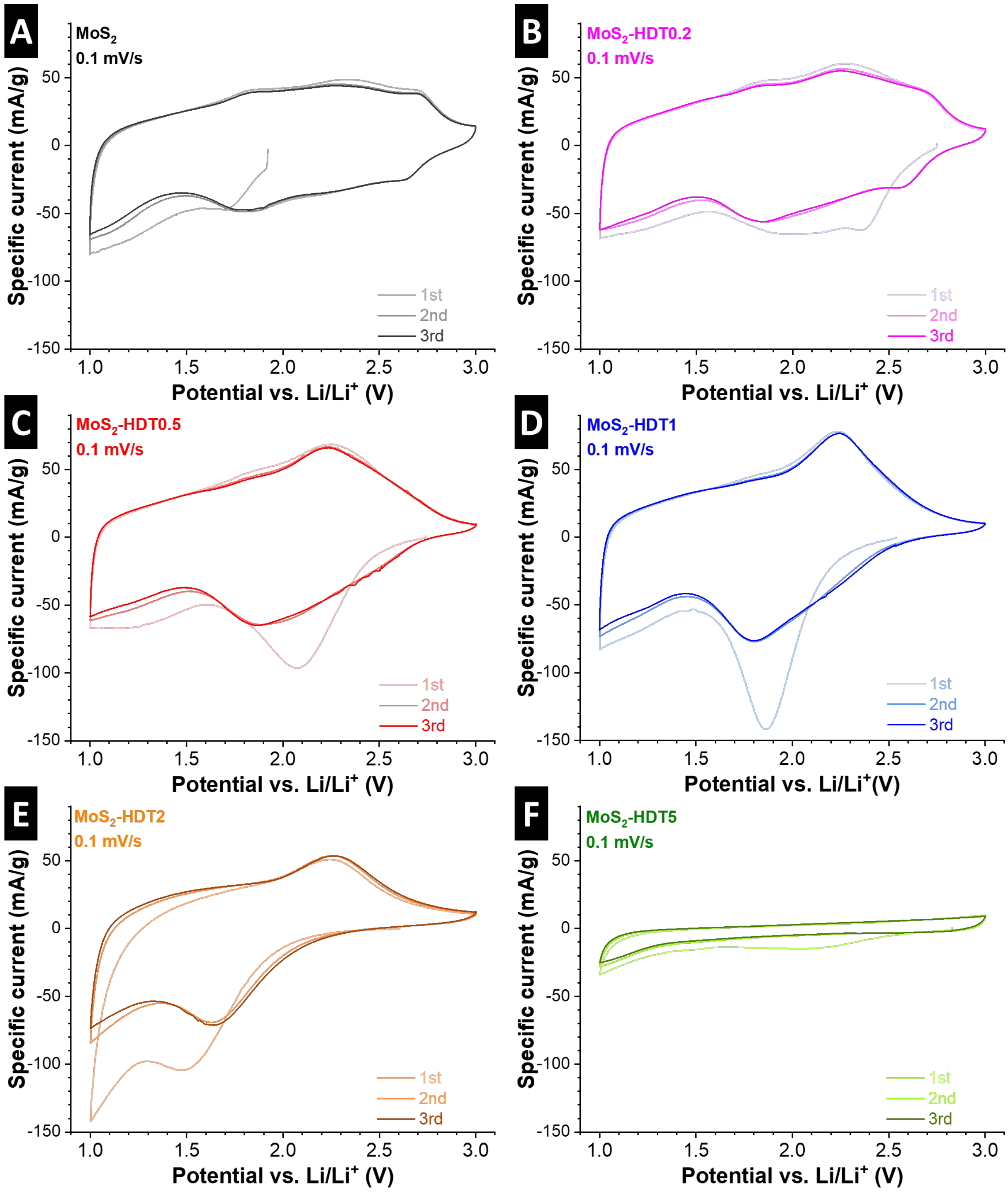

Fig. S8: Cyclic voltammograms at 0.1 mV/s of first three cycles of all MoS_2-based samples. Recorded in coin cells in LP30 electrolyte at a constant temperature of 20 °C.

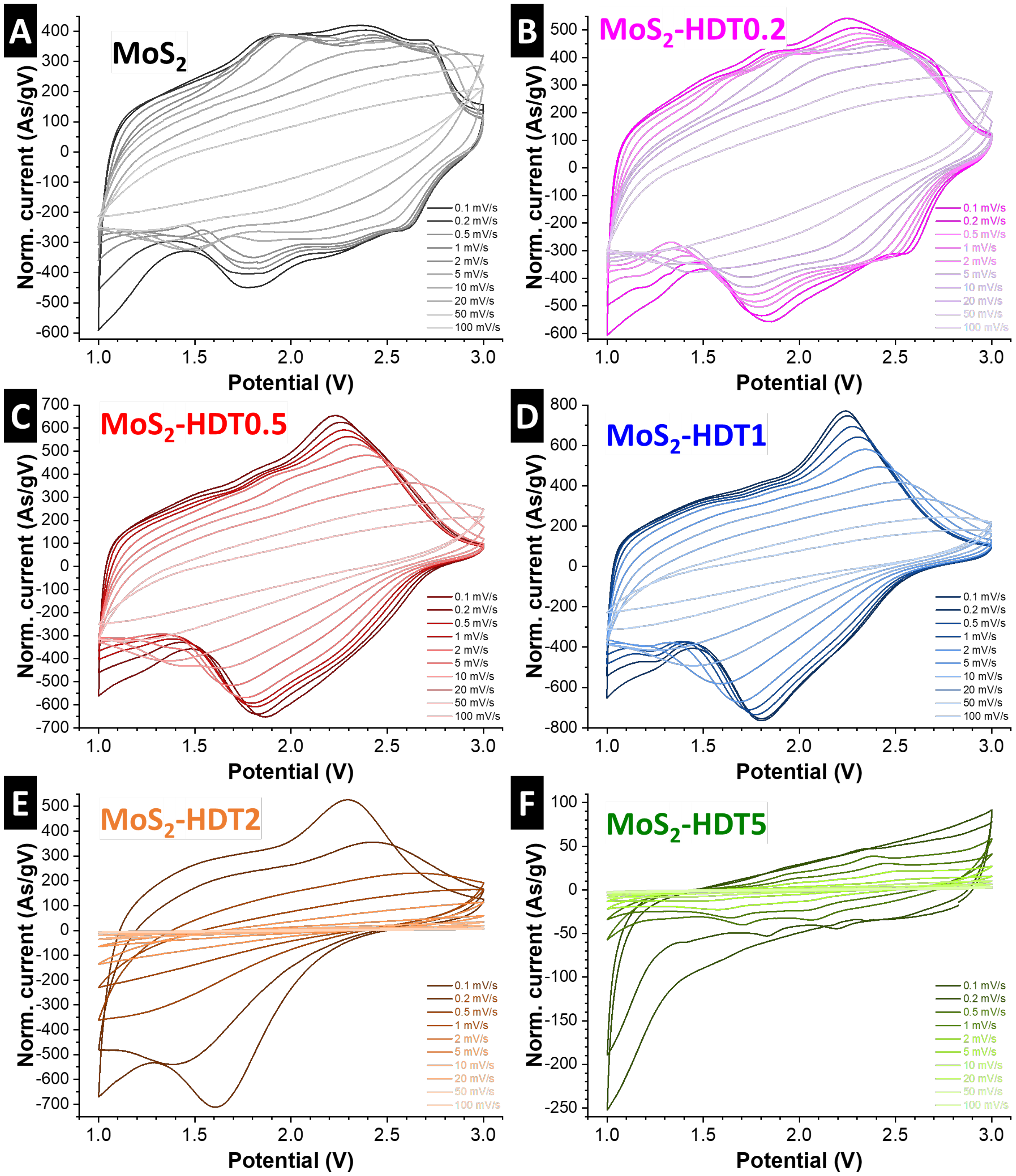

Fig. S9: Cyclic voltammograms of all MoS_2-based samples at sweep rates of 0.1, 0.2, 0.5, 1, 2, 5, 10, 20, 50, and 100 mV/s. Current is normalized by scan rate for better visibility of low sweep rates. Recorded in coin cells in LP30 electrolyte at a constant temperature of 20 °C.

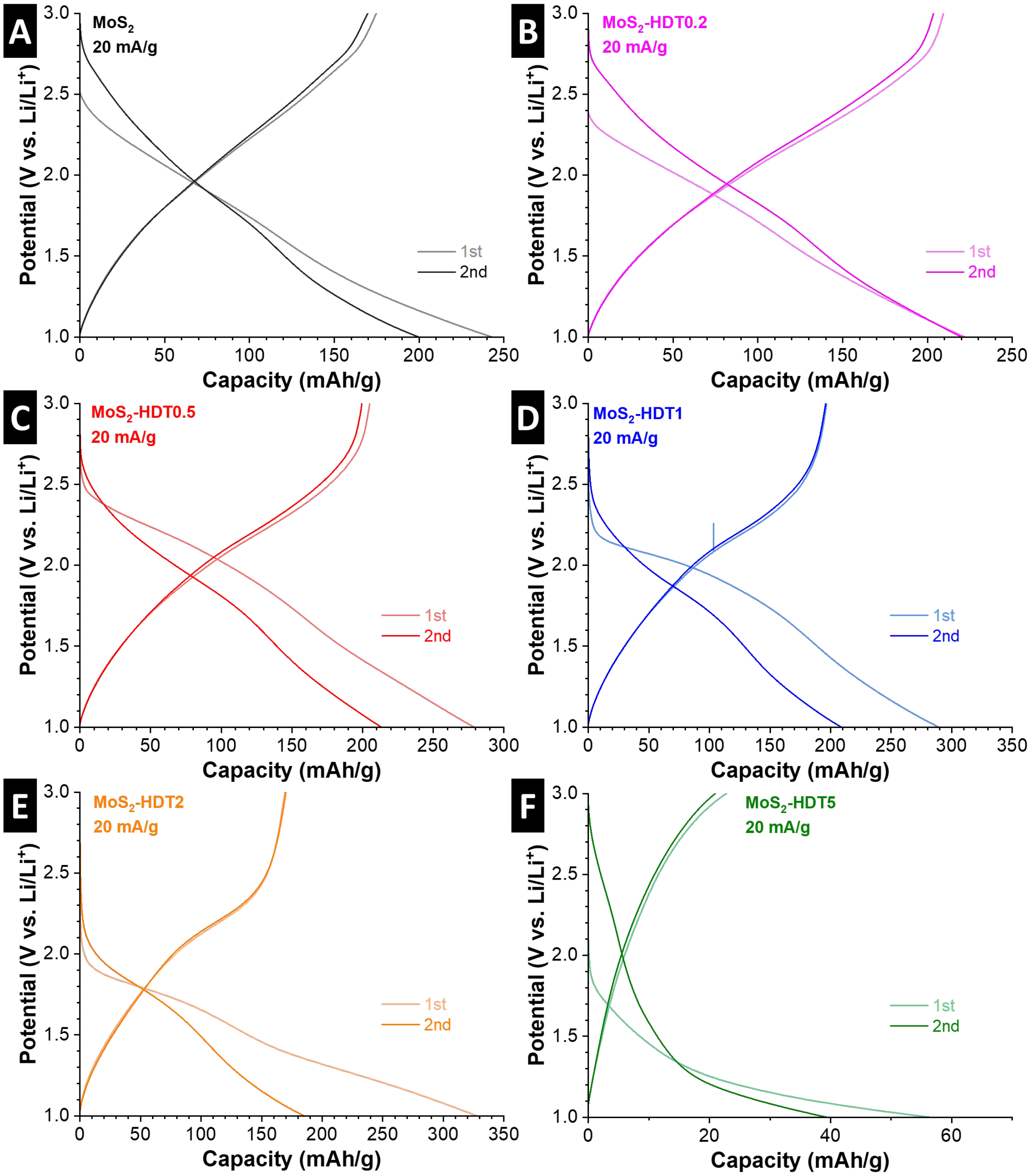

Fig. S10: First two galvanostatic charge/discharge of all MoS2-based samples at a specific current of 20 mA/g. Recorded in coin cells in LP30 electrolyte at a constant temperature of 20 °C.

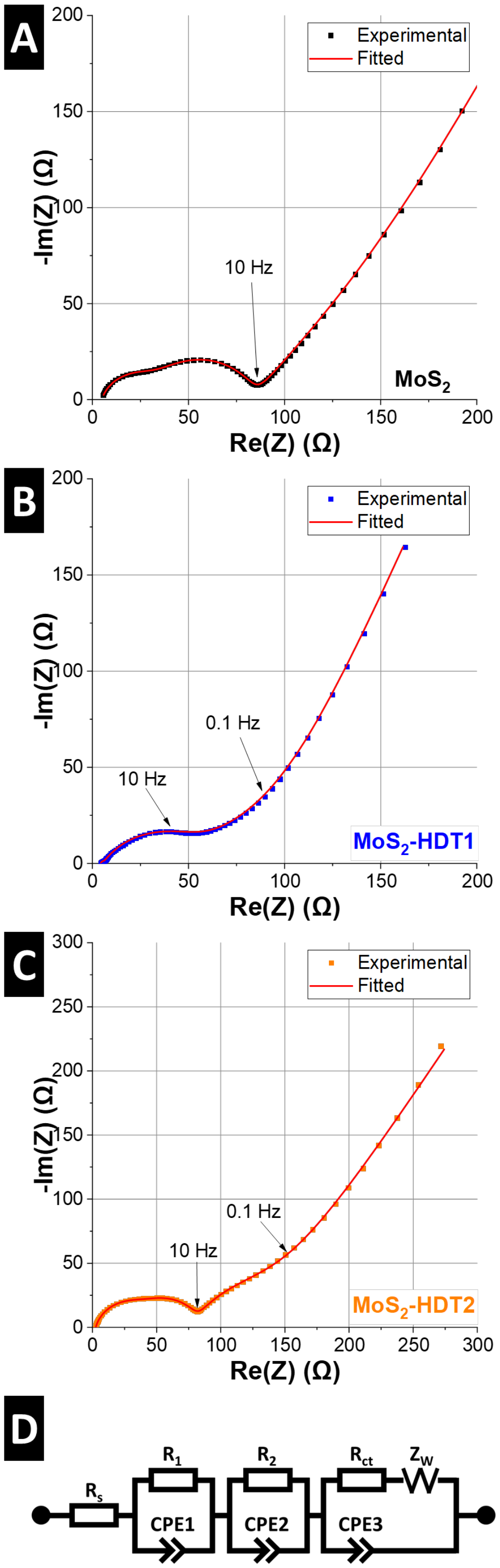

Fig. S11: Electrochemical impedance spectra of (A) MoS_2, (B) MoS_2-HDT1, and (C) MoS_2-HDT2 at 1.2 V vs. Li^+/Li. The data is fitted using the equivalent circuit shown in (D). Rs corresponds to the ohmic resistance of the electrochemical cell, the first RQ-element is to correct for high frequency artifacts from the counter electrode, the second RQ-element corresponds to surface film impedance, Rct is the charge transfer resistance and Zw is an open Warburg element corresponding to finite-length diffusion.

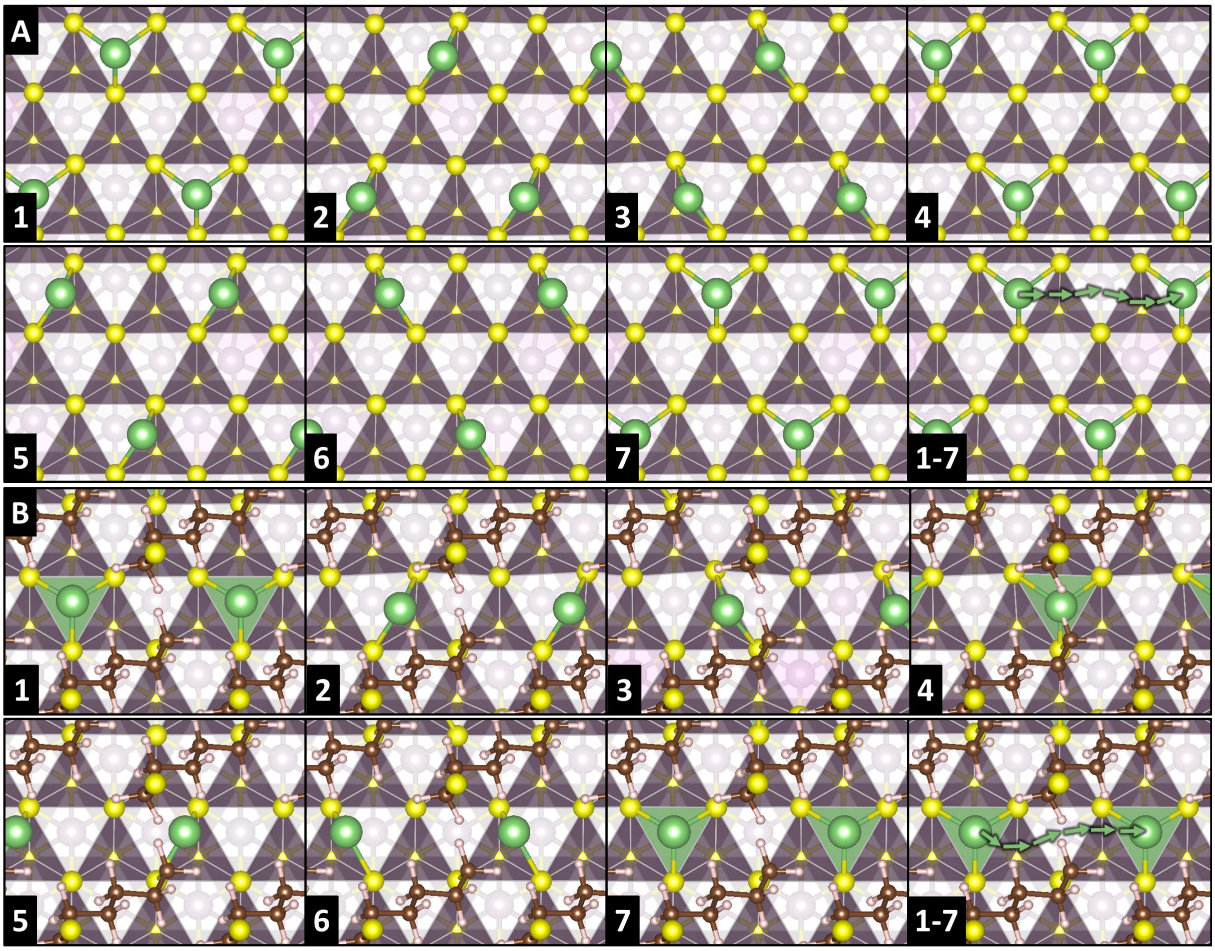

Fig. S12: The top view of the complete trajectory for each CI-NEB simulation is presented for (A) pristine MoS_2 and (B) MoS_2-HDT. The pillar restructuring is observed in image **B3**.
